# Development and Validation of a 9-Gene Prognostic Signature in Patients With Multiple Myeloma

**DOI:** 10.3389/fonc.2018.00615

**Published:** 2019-01-08

**Authors:** Xiao-Ping Liu, Xiao-Hong Yin, Xiang-Yu Meng, Xin-Hui Yan, Fan Wang, Li He

**Affiliations:** ^1^Zhongnan Hospital of Wuhan University, Wuhan, China; ^2^Department of Cardiology, the First Hospital of Lanzhou University, Lanzhou, China; ^3^Department of Gastroenterology, Zhongnan Hospital of Wuhan University, Wuhan, China; ^4^Department of Hematology, Zhongnan Hospital of Wuhan University, Wuhan, China

**Keywords:** multiple myeloma, weighted co-expression network analysis, prognostic signature, LASSO Cox proportional hazards regression model, survival

## Abstract

**Background:** Multiple myeloma (MM) is one of the most common types of hematological malignance, and the prognosis of MM patients remains poor.

**Objective:** To identify and validate a genetic prognostic signature in patients with MM.

**Methods:** Co-expression network was constructed to identify hub genes related with International Staging System (ISS) stage of MM. Functional analysis of hub genes was conducted. Univariate Cox proportional hazard regression analysis was conducted to identify genes correlated with the overall survival (OS) of MM patients. Least absolute shrinkage and selection operator (LASSO) penalized Cox proportional hazards regression model was used to minimize overfitting and construct a prognostic signature. The prognostic value of the signature was validated in the test set and an independent validation cohort.

**Results:** A total of 758 hub genes correlated with ISS stage of MM patients were identified, and these hub genes were mainly enriched in several GO terms and KEGG pathways involved in cell proliferation and immune response. Nine hub genes (HLA-DPB1, TOP2A, FABP5, CYP1B1, IGHM, FANCI, LYZ, HMGN5, and BEND6) with non-zero coefficients in the LASSO Cox regression model were used to build a 9-gene prognostic signature. Relapsed MM and ISS stage III MM was associated with high risk score calculated based on the signature. Patients in the 9-gene signature low risk group was significantly associated with better clinical outcome than those in the 9-gene signature high risk group in the training set, test, and validation set.

**Conclusions:** We developed a 9-gene prognostic signature that might be an independent prognostic factor in patients with MM.

## Introduction

Multiple myeloma (MM), originated from malignant plasma cells secreting monoclonal M protein, represents the second most common malignancy in hematological malignancies (1–3). MM is differentiated from monoclonal gammopathy of undetermined significance (MGUS) and smoldering multiple myeloma (SMM) by the presence of end-organ damage (4, 5). The clinical symptoms of MM range from asymptomatic forms to manifestations of anemia, bone pain, and eventually spontaneous of fractures, renal failure, and frequent infections (6–8). Thanks to the introduction of novel agents (proteasome inhibitors, immunomodulatory drugs, and monoclonal antibodies), the management strategies for MM have been improved considerably in the past decade (9–12). Accordingly, the clinical outcomes of patients with MM have been significantly improved, however, MM remains an incurable disease and the prognosis of patients with MM remains poor (with a median survival of approximately 3–4 years) (13).

The International Staging System (ISS) stage of MM, based on β_2_-microglobulin (β_2_M) and albumin (ALB), divides MM patients into three different stages with significant dissimilar clinical outcomes. The ISS was a collaborative effort by investigators from 17 institutions worldwide and from data on 11,171 patients (4, 14). Patients with stage 1, 2, 3 diseases have median survivals of 62, 44, 29 months, respectively (4, 15).

Weighted gene co-expression network analysis (WGCNA), a systems biology algorithm that can be applied to describing the correlation patterns among genes across microarray samples, finding and summarizing modules of high related genes, and relating modules to certain clinical phenotype (16, 17), is widely used to facilitate the screening or identification of candidate biomarkers or therapeutic targets (18). Therefore, in the present study, we used WGCNA to screen potentially relevant molecular biomarkers correlated with the ISS stages of patients with MM. Moreover, we developed and validated the associated signature in patients with MM.

## Methods

### MM Microarray Data

MM gene expression data and clinical information were downloaded from gene expression omnibus (GEO) database (https://www.ncbi.nlm.nih.gov/geo/) and ArrayExpress (http://www.ebi.ac.uk/arrayexpress/). Affymetrix gene expression profiles were performed using Affymetrix Human Genome U133 Plus 2.0 (HG-U133 Plus_2.0) [GSE19784 (19) and GSE24080 (20)], Affymetrix Human Genome U133A Array(GSE6477) (21, 22), and Affymetrix GeneChip Human Gene 1.0 ST Array[E-MTAB-4032 (23)]. GSE19784, including 328 samples from patients with newly diagnosed MM, was used to construct the co-expression network. GSE24080, including 559 untreated MM samples, was randomly assigned patients in a 3:2 ratio to a training set and test set to develop and validate a prognostic signature. GSE6477, including 15 samples of normal donors, 73 samples of newly diagnosed MM, and 28 samples of relapsed MM, was used to evaluate the risk score calculated based on the prognostic signature among normal donor, newly diagnosed MM, and relapsed MM. E-MTAB-4032 (including 151 untreated MM) was used as an independent validation cohort to evaluate the prognostic role of the signature. Raw data of GSE19784 and E-MTAB-4032 were preprocessed using the R/Bioconductor “affy” package (24) and oligo (25) package, respectively. Robust Multi-array Average (RMA) (26) normalized data of these two studies at gene level were analyzed. For GSE6477 and GSE24080, the raw data had been normalized using MAS5 method and the expression levels of genes were transformed using the logarithm function. Purified CD138+ plasma cells (including myeloma cells and normal plasma cells) in GSE19784, GSE6477, E-MTAB-4032, and GSE20480 were separated using positive magnetic cell sorting selection with CD138 magnetic microbeads and subjected to gene expression profiling (GEP) as mentioned previously (19–23).

### Construction of Co-expression Network and Identification of Hub Genes

The R package “WGCNA” (27) was used to construct a co-expression network for genes with highest variances (top 10000) in GSE19784. Prior to constructing the co-expression network, we applied sample networks method which was introduced by Oldham et al to detect outliers (28). A sample was considered as outlying, if the associated Z.K value was <-2.5. The soft threshold power β was selected according to the scale-free topology criterion as introduced previously (16, 17). Subsequently, Pearson's correlations between each gene pair was calculated to generate a matrix of adjacencies, and then the adjacencies were transformed into topological overlap matrix (TOM) (29). Next, we conducted average linkage hierarchical clustering based on the TOM-based dissimilarity. The minimum module size and medium sensitivity was 30 and 2, respectively, and other parameters were default. After relating modules to the ISS stage of MM patients and calculating the associated Gene Significance (the correlation between the genes and the trait) and Module Membership (the correlation of the first principal component of the expression matrix of the corresponding module and the gene expression profile), we screened hub genes using a networkScreening function based on Gene Significance and Module Membership and genes with q.Weighetd value (q-value (local FDR) calculated from weighted *p*-value of association with the ISS stage of MM) < 0.01 were finally treated as hub genes (30).

### Functional Enrichment Analysis of Hub Genes

To understand the biological function of the hub genes, we performed gene ontology (GO), and Kyoto Encyclopedia of Genes and Genomes (KEGG) enrichment analysis using the DAVID (31) online tool. GO and KEGG terms at *P* < 0.05 and false discovery rate (FDR) < 0.05 were considered significantly enriched and the significantly enriched GO and KEGG terms were visualized using R package “ggplot2” (32).

### Development of the Prognostic Signature Based on the Hub Genes

To investigate the associations between the hub genes and the survival of MM patients, we performed univariate Cox proportional hazards regression model in GSE24080. Genes significantly correlating with the overall survival (OS) of MM patients were included in a Least absolute shrinkage and selection operator (LASSO) penalized Cox proportional hazards regression model to minimize overfitting, and a 10-fold cross validation was also conducted using the R package glmnet (33, 34). Then, we calculated the risk score for each patient based on this penalized Cox proportion model in the training set.

### Validation of the Predictive Value of the Prognostic Signature in MM Patients

To validate the predictive value of the prognostic signature, Kaplan-Meier survival analysis, and univariate and multivariable Cox proportional hazards regression model were performed in the training set and test set in terms of OS, and event-free survival (EFS). Prior to multivariable Cox proportional hazards regression analysis on the OS, and EFS, we performed a variable selection based on the LASSO penalized Cox proportional hazards regression model. The definitions of OS and EFS was introduced previously (21–23). Meanwhile, we also validated the performance of the signature in the independent cohort E-MTAB-4032. The above survival analyses were conducted using the R packages “survival” (35) and “survminer” (version 0.4.3). MM patients in GSE24080 and E-MTAB-4032 were classified into the prognostic low risk group and the 9-signature high risk group based on the cutoff calculated through time dependent receiver operating characteristic (ROC) analysis using the R package “survivalROC” (36). The risk score of the signature in patients with ISS I, II, and III disease were evaluated using E-MTAB-4032. Meanwhile, the risk score of the signature in normal plasma cells, untreated MM, and relapsed MM were evaluated using GSE6477. The risk scores of the signature in E-MTAB-4032 and GSE6477 were presented as mean ± the standard error of the mean (SEM). Grouped data was analyzed using unpaired *T*-test, and *P* < 0.05 was considered statistically significant.

## Results

### Results of Data Preprocessing, Co-expression Network Construction and Hub Genes Identification

No sample was demonstrated to be an outlier after all samples were clustered based on their Euclidean distances. Meanwhile, β = 12, the lowest power for which the scale-free topology fit index reaches 0.9, was used for the subsequent adjacency calculation. After TOM based clustering, 14 gene modules were obtained. After co-expression network construction, a total of 780 probes were identified based on our screening criteria, 758 of which annotated to gene symbol were treated as hub genes (Supplementary Table 1). The major process of co-expression network construction and hub gene identification was shown in Supplementary Figure 1.

### GO and KEGG Pathway Enrichment Analysis of Hub Genes

**Figure 1:**
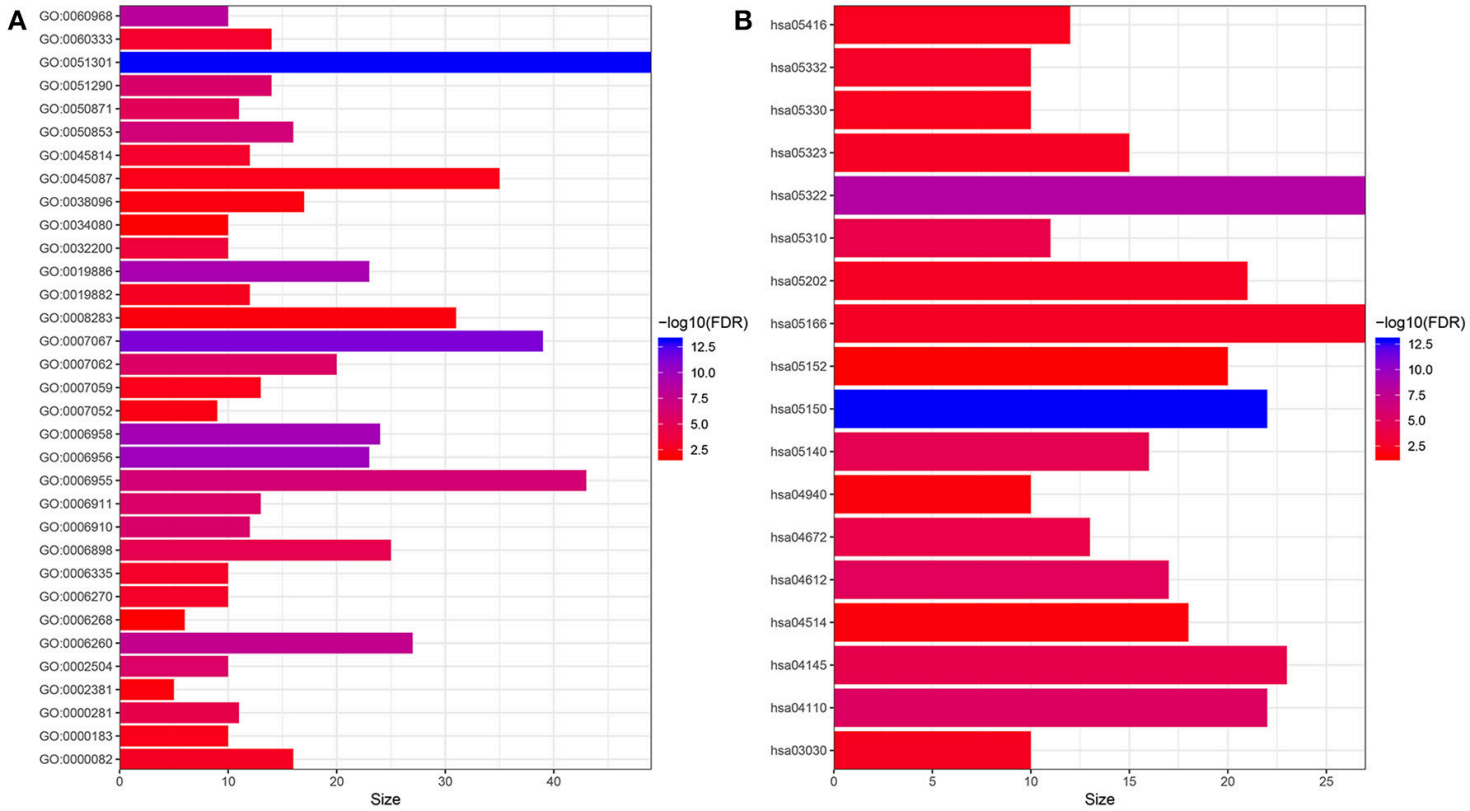
Functional enrichment analysis of hub genes. **(A)** GO enrichment analysis. **(B)** KEGG enrichment analysis. GO:0051301~cell division, GO:0007067~mitotic nuclear division, GO:0006956~complement activation, GO:0006958~complement activation, classical pathway, GO:0019886~antigen processing and presentation of exogenous peptide antigen via MHC class II, GO:0060968~regulation of gene silencing, GO:0006260~DNA replication, GO:0050853~B cell receptor signaling pathway, GO:0006955~immune response, GO:0051290~protein heterotetramerization, GO:0006910~phagocytosis, recognition, GO:0006911~phagocytosis, engulfment, GO:0002504~antigen processing and presentation of peptide or polysaccharide antigen via MHC class II, GO:0007062~sister chromatid cohesion, GO:0050871~positive regulation of B cell activation, GO:0006898~receptor-mediated endocytosis, GO:0000281~mitotic cytokinesis, GO:0032200~telomere organization, GO:0060333~interferon-gamma-mediated signaling pathway, GO:0045814~negative regulation of gene expression, epigenetic, GO:0006335~DNA replication-dependent nucleosome assembly, GO:0006270~DNA replication initiation, GO:0000082~G1/S transition of mitotic cell cycle, GO:0019882~antigen processing and presentation, GO:0007059~chromosome segregation, GO:0000183~chromatin silencing at rDNA, GO:0045087~innate immune response, GO:0007052~mitotic spindle organization, GO:0038096~Fc-gamma receptor signaling pathway involved in phagocytosis, GO:0008283~cell proliferation, GO:0002381~immunoglobulin production involved in immunoglobulin mediated immune response, GO:0006268~DNA unwinding involved in DNA replication, GO:0034080~CENP-A containing nucleosome assembly; hsa05150:Staphylococcus aureus infection, hsa05322:Systemic lupus erythematosus, hsa04110:Cell cycle, hsa04612:Antigen processing and presentation, hsa05140:Leishmaniasis, hsa04145:Phagosome, hsa05310:Asthma, hsa04672:Intestinal immune network for IgA production, hsa05332:Graft-vs.-host disease, hsa05166:HTLV-I infection, hsa05202:Transcriptional misregulation in cancer, hsa05323:Rheumatoid arthritis, hsa03030:DNA replication, hsa05330:Allograft rejection, hsa05416:Viral myocarditis, hsa04940:Type I diabetes mellitus, hsa04514:Cell adhesion molecules (CAMs), hsa05152:Tuberculosis.

In order to have a preliminary understanding of the biological significance, we conducted GO and KEGG enrichment analysis. As shown in Figure 1, the hub genes were mostly enriched in GO terms related to cell proliferation (“cell division,” “cell proliferation,” “mitotic nuclear division,” “DNA replication,” “DNA unwinding involved in DNA replication,” “sister chromatid cohesion,” “mitotic cytokinesis,” “DNA replication-dependent nucleosome assembly,” “DNA replication initiation,” “G1/S transition of mitotic cell cycle,” “chromosome segregation,” and “mitotic spindle organization”) and immune response (“complement activation,” “antigen processing and presentation of exogenous peptide antigen via MHC class II,” “ B cell receptor signaling pathway,” “ immune response,” “phagocytosis, recognition,” “ positive regulation of B cell activation,” “receptor-mediated endocytosis,” “interferon-gamma-mediated signaling pathway,” “innate immune response,” “Fc-gamma receptor signaling pathway involved in phagocytosis,”and “immunoglobulin production involved in immunoglobulin mediated immune response”) (Figure 1A). Furthermore, the results of KEGG pathway enrichment analysis of the hub genes suggested that these genes were mainly enriched in infection or immune related pathways (“Staphylococcus aureus infection,” “antigen processing and presentation,” “leishmaniasis,” “asthma,” “intestinal immune network for IgA production,” “graft-vs.-host disease,” “HTLV-I infection,”and “Rheumatoid arthritis”), and cell proliferation (“cell cycle,”and “DNA replication”) (Figure 1B).

### Development of a 9-Gene Signature in Patients With MM

To investigate the prognostic value of the hub genes, we conducted univariated Cox proportional hazards regression analysis, and the results suggested that the expression of 325 hub genes were significantly correlated with the OS of MM patients in the training set of GSE24080. To avoid overfitting as much as possible, we conducted LASSO penalized Cox proportional hazards regression model in the training set in GSE24080, and the results identified 9 of the 325 hub genes (HLA-DPB1(major histocompatibility complex, class II, DP beta 1), TOP2A (topoisomerase 2A), FABP5 (Fatty Acid-Binding Protein 5), CYP1B1(cytochrome P450 family 1 subfamily B member 1), IGHM (immunoglobulin heavy constant mu), FANCI (FA complementation group I), LYZ (lysozyme), HMGN5 (high mobility group protein N5 subtype), and BEND6 (BEN domain containing 6) with non-zero coefficient. Thus, we built the 9-gene signature on the basis of the coefficients of these 9 hub genes in the LASSO penalized model (Supplementary Table 2).

### Associations Between the 9-Gene Signature and the ISS Stage of MM and the Disease Status

**Figure 2:**
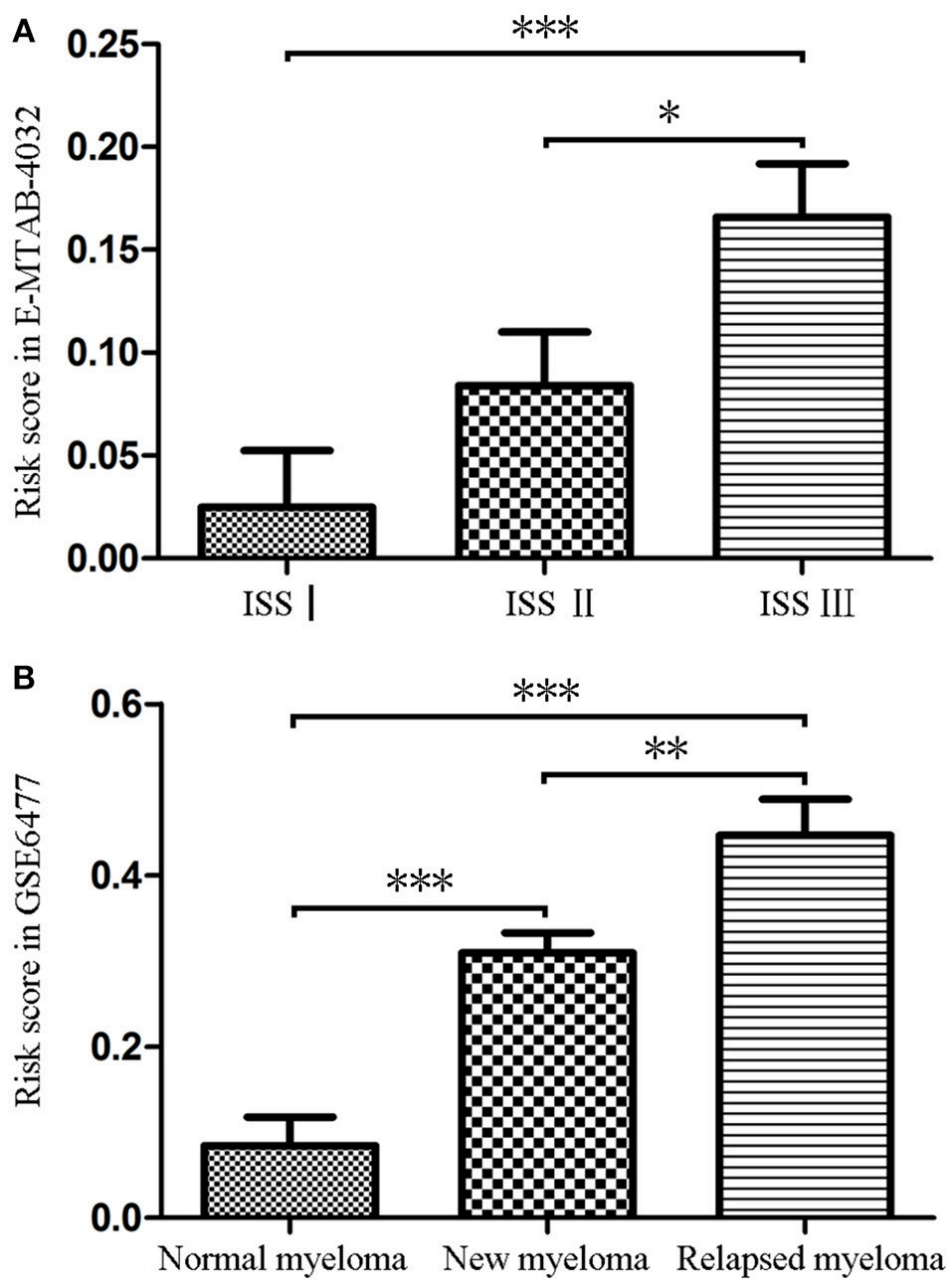
The risk score of the signature in GSE6477 and E-MTAB-4032. **(A)** The risk score was increased in MM patients with ISS III disease and ISS IIcompared with that in MM patients with ISS Idisease. **(B)** The risk score of was significantly upregulated in newly diagnosed MM and relapsed MM compared with that in normal plasma cells. ^*^P < 0.05, ^**^P < 0.01, and ^***^P < 0.001.

Firstly, we calculated the risk score of each MM patients for the 9-gene signature in GSE6477 and E-MTAB-4032 based on the coefficients of the 9 hub genes (Supplementary Table 2). As shown in Figure 2A, the risk score of patients with ISS stage III disease was significantly higher than that of patients with ISS stageI (*t* = −0.362, *P* = 0.001) and II (*t* = −0.218, *P* = 0.031). Meanwhile, the risk score of patients with relapsed MM was significantly higher compared with that of normal donor (*t* = 5.782, *P* < 0.001) and patients with untreated MM (*t* = 2.977, *P* = 0.004), and the risk score were higher in patients with untreated MM compared with that in normal donor (*t* = −4.13, *P* < 0.001, Figure 2B).

### Evaluation the Prognostic Role of the 9-Gene Signature

**Figure 3:**
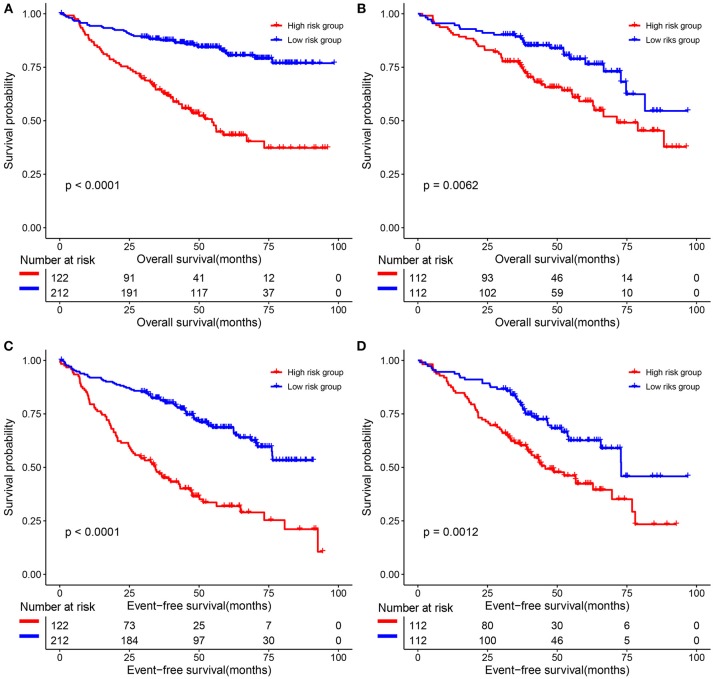
The correlations between the 9-gene signature and the overall survival (OS) and event-free survival (EFS) of patients with MM. **(A)** OS in the training set. **(B)** OS in the test set. **(C)** EFS in the training set. **(D)** EFS in the test set.

**Figure 4:**
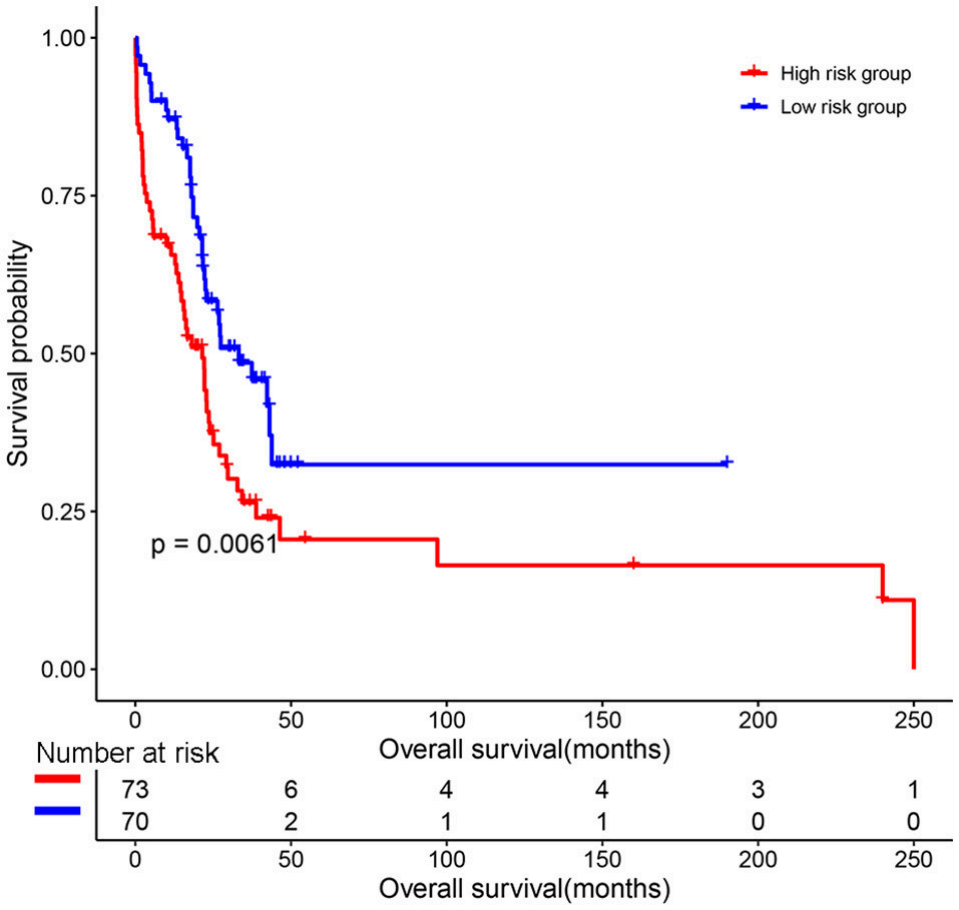
The prognostic role of the 9-gene signature in the independent validation cohort E-MTAB-4032.

The predictive value of the 9-gene signature was evaluated in the training set, test set and an independent set E-MTAB-4032. Based on the optimal cutoff (1.939) calculated using time dependent ROC analysis (Supplementary Figure 1). The Kaplan-Meier survival analysis suggested that patients in the 9-gene signature low risk group had better OS compared with those in the 9-gene signature high risk group in the training set (HR = 0.2664, 95% CI: 0.1772-0.4007, log-rank *P* < 0.001, Figure 3A, Supplementary Table 3) and the test set (HR = 0.5115, 95% CI: 0.3137–0.8339, log-rank *P* = 0.0062, Figure 3B, Supplementary Table 4). Meanwhile, patients in the 9-gene signature low risk group had with better EFS compared with those in the 9-gene signature high risk group in the training set (HR = 0.3321, 95% CI:0.2395–0.4606, *P* < 0.0001, Figure 3C, Supplementary Table 5) and test set (HR = 0.5174, 95% CI: 0.3447–0.7765, *P* = 0.0015, Figure 3D, Supplementary Table 6). Based on the results of variable selection (Supplementary Table 7), age, B2M (β2-microglobin), CRP (C reaction protein), LDH (lactate dehydrogenase), BMPC (bone marrow plasma cell), MRI (magnetic resonance imaging), and the 9-gene signature was included multivariable Cox proportional hazards regression analysis which indicated that the 9-gene signature was an independent prognostic factor in terms of OS and EFS in the training set and test (Supplementary Tables 3–6). Moreover, MM patients in the 9-gene low risk group also had better OS compared with those in the 9-gene signature high risk group in the independent validation cohort E-MTAB-4032 (HR = 10.6091, 95% CI: 3.2120–35.0409, log-rank *P* = 0.0061, Figure 4). Meanwhile, the results of multivariable Cox proportional hazards suggested that the 9-gene signature was also an independent prognostic factor in the validation cohort (HR = 14.8092, 95% CI:1.2282–178.5591, *P* = 0.0339, Supplementary Table 7).

## Discussion

As stated above, although several novel agents for patients with MM have been introduced into clinical practice, the disease remained incurable, and the clinical outcome of patients with MM is still poor (2, 3). Thus, it is of vital importance to develop such molecular biomarkers or signature that are significantly correlated with the clinicopathological features and clinical outcome of patients with MM. In the present study, a total of 758 hub genes associated with the ISS stage of MM patients were identified through WGCNA. Thus, we performed univariate Cox proportional hazards regression analysis to analyzed the relations between the expression of hub genes and the OS of patients with MM, and the results suggested that 325 hub genes were associated with the OS of MM patients. LASSO (34) was introduced in order to improve the prediction accuracy and interpretability of regression models by forcing the sum of the absolute value of the regression coefficients to be less than a fixed value, which forces certain coefficients to be set to zero, effectively choosing a simpler model that does not include those coefficients. Based on this, we included the above 325 hub genes into a LASSO penalized Cox proportional hazards regression model, as a result, 9 hub genes with none-zero coefficients in this model was identified. Thus, we calculated the risk score of each MM patients, and built the 9 hub genes [HLA-DPB1(major histocompatibility complex, class II, DP beta 1), TOP2A (topoisomerase 2A), FABP5 (Fatty Acid-Binding Protein 5), CYP1B1(cytochrome P450 family 1 subfamily B member 1), IGHM (immunoglobulin heavy constant mu), FANCI (FA complementation group I), LYZ (lysozyme), HMGN5 (high mobility group protein N5 subtype), and BEND6 (BEN domain containing 6)] based signature.

Results of functional enrichment analysis of hub genes suggested that hub genes were mainly enriched in cell proliferation and immune response related GO terms and pathways, this was in accordance with the prognostic value of the 9-gene signature developed based on the hub genes. MM patients in the 9-gene low risk group was associated with better OS and EFS compared with those in the 9-gene high risk group, and the 9-gene signature was shown to be an independent prognostic signature in patients with MM.

Actually, most of these hub genes in the signature had been demonstrated be associated with the proliferation or invasion of several human cancers. HLA-DPB1, also known as major histocompatibility complex, class II, DP beta 1, belongs to the HLA-class IIbeta chain paralogue. Li et al. demonstrated that genetic variant of HLA-DPB1 increased in the risk of extranodal natural killer T-cell lymphoma (37). Liu et al. demonstrated that TOP2A (topoisomerase 2A) and TOP1 functioned as oncogene in liver cancer (38). Meanwhile, decreased expression of TOP2A inhibited the proliferation of invasion of colon cancer cells (39). Kawaguchi et al and Powell et al. demonstrated that overall expression of FABP5 promoted the proliferation and metastasis of colorectal cancer cells and breast cancer cells (40). Gu et al. demonstrated that genetic variant of CYP1B1 gene was associated prognosis of patients with prostate cancer (41). Mutation patterns of IGHM was associated different progression pathways in follicular lymphoma (42). Chen et al demonstrated that PRC1 could promote early recurrence of patients with hepatocellular carcinoma by regulating the expression of FANCI (43). Mariano FV et al demonstrated that the expression of LYZ could be used to differentiate mammary analog secretory carcinoma from acinic cell carcinoma of salivary glands (44). Wu et al demonstrated that the expression of HMGN5 was increased in bladder cancer cells and high expression of HMGN5 was associated with poor prognosis of patients with bladder cancer (45). The results of the above literature review provided strong support for the 9-gene signature in the clinical out prediction in patients with MM.

Limits of our study are as follows. First, our study is a retrospective analysis based on previously published MM gene expression studies, although its conclusions have been confirmed in the test set and independent validation set, we recommend that the conclusions of this study should be verified by molecular biology experiments in subsequent studies. Second, the prognostic performance of the 9-gene signature should be evaluated through prospective clinical trials.

Taken together, we developed a 9-gene prognostic signature based on the hub genes obtained through a co-expression network, patients in the 9-gene signature low risk group were associated with better clinical outcomes compared with those in the 9-gene signature high risk group.

## Author Contributions

X-PL collected and analyzed the data, write the manuscript. Xia-HY analyzed the data, and review the manuscript. X-YM and Xin-HY participated in revising the manuscript. FW revised the manuscript, analyzed the data, and participated in preparation of the figures. LH designed the study and participated in data analysis.

### Conflict of Interest Statement

The authors declare that the research was conducted in the absence of any commercial or financial relationships that could be construed as a potential conflict of interest.

## Supplementary Material

Supplementary Figure 1 Flowchart depicting the major process of Co-expression network construction and identification of hub genes.

Supplementary Figure 2 Time-Dependent ROC Curve for the 9-gene signature in the training set at 90 months.

Supplementary Table 1 Hub genes calculated using weighted co- expression network analysis and their impact on the overall survival of patients with multiple myeloma.

Supplementary Table 2 The 9 hub genes with non-zero coefficients in the LASSO Cox proportional hazards regression model.

Supplementary Table 3 Cox proportional hazards regression analysis the association between the clinical characteristics and overall survival of multiple myeloma in the training set.

Supplementary Table 4 Cox proportional hazards regression analysis on the association between the clinical characteristics and overall survival of multiple myeloma patients in the test. set.

Supplementary Table 5 Cox proportional hazards regression analysis on the association between the clinical characteristics and event-free survival of multiple myeloma patients in the training set.

Supplementary Table 6 Cox proportional hazards regression analysis on the association between the clinical characteristics and event-free survival of multiple myeloma patients in the test set.

Supplementary Table 7 The prognostic role of the 9-gene signature in the independent validation cohort.

## References

[1] Merz M Kellermann L Poenisch W Tischler HJ Kohnke J Knauf W. Diagnosis and treatment of multiple myeloma in Germany: analysis of a nationwide multi-institutional survey. Ann Hematol. (2017) 96:987–93. 10.1007/s00277-017-2991-0 28409228

[2] Gozzetti A Papini G Defina M Bocchia M. Extramedullary myeloma relapses. Ann Hematol. (2012) 91:1511–2. 10.1007/s00277-012-1432-3 22362123

[3] Chan HSH Chen CI Reece DE. Current review on high-risk multiple myeloma. Curr Hematol Malig Rep. (2017) 12:96–108. 10.1007/s11899-017-0368-z 28317082

[4] Rajkumar SV Buadi F. Multiple myeloma: new staging systems for diagnosis, prognosis and response evaluation. Best Pract Res Clin Haematol. (2007) 20:665–80. 10.1016/j.beha.2007.10.002 18070712

[5] Bataille R Annweiler C Beauchet O. Multiple myeloma international staging system: “staging” or simply “aging” system? Clin Lymphoma Myeloma Leuk (2013) 13:635–7. 10.1016/j.clml.2013.07.003 24035714

[6] MacLennan IC Drayson M Dunn J. Multiple myeloma. BMJ (1994) 308:1033–6. 10.1136/bmj.308.6935.1033 8068084 PMC2539886

[7] Bergsagel PL Where we were, where we are, where we are going: progress in multiple myeloma. Am Soc Clin Oncol Educ Book (2014) 2014:199–203. 10.14694/EdBook_AM.2014.34.199 24857077

[8] Bergin K McQuilten Z Moore E Wood E Spencer A. Myeloma in the real world: what is really happening? Clin Lymphoma Myeloma Leuk (2017) 17:133-144 e131. 10.1016/j.clml.2016.12.002 28153487

[9] Liu X Chen J He YA Meng X Li K He CK. Comparing efficacy and survivals of initial treatments for elderly patients with newly diagnosed multiple myeloma: a network meta-analysis of randomized controlled trials. Onco Targets Ther. (2017) 10:121–8. 10.2147/OTT.S123680 28053546 PMC5189699

[10] Berenson A Vardanyan S David M Wang J Harutyunyan NM Gottlieb J. Outcomes of multiple myeloma patients receiving bortezomib, lenalidomide, and carfilzomib. Ann Hematol. (2017) 96:449–59. 10.1007/s00277-016-2889-2 27933373

[11] Terpos E International Myeloma S. Multiple myeloma: clinical updates from the american society of hematology annual meeting 2016. Clin Lymphoma Myeloma Leuk (2017) 17:329–39. 10.1016/j.clml.2017.02.010 28462890

[12] Cejalvo MJ de la Rubia J. Which therapies will move to the front line for multiple myeloma? Exp Rev Hematol. (2017) 10:383–92. 10.1080/17474086.2017.1317589 28388244

[13] Cejalvo MJ de la Rubia J. Clinical treatment of newly diagnosed multiple myeloma. Expert Rev Hematol. (2015) 8:595–611. 10.1586/17474086.2015.1078236 26327587

[14] Greipp PR San Miguel J Durie BG Crowley JJ Barlogie B Blade J. International staging system for multiple myeloma. J Clin Oncol. (2005) 23:3412–20. 10.1200/JCO.2005.04.242 15809451

[15] Kyrtsonis MC Maltezas D Tzenou T Koulieris E Bradwell AR. Staging systems and prognostic factors as a guide to therapeutic decisions in multiple myeloma. Semin Hematol. (2009) 46:110–7. 10.1053/j.seminhematol.2009.02.004 19389494

[16] Zhang B Horvath S. A general framework for weighted gene co-expression network analysis. Stat Appl Genet Mol Biol. (2005) 4:17. 10.2202/1544-6115.1128 16646834

[17] Yip AM Horvath S. Gene network interconnectedness and the generalized topological overlap measure. BMC Bioinformatics (2007) 8:22. 10.1186/1471-2105-8-22 17250769 PMC1797055

[18] Li S Liu X Liu T Meng X Yin X Fang C. Identification of Biomarkers Correlated with the TNM Staging and Overall Survival of Patients with Bladder Cancer. Front Physiol. (2017) 8:947. 10.3389/fphys.2017.00947 29234286 PMC5712410

[19] Broyl A Hose D Lokhorst H de Knegt Y Peeters J Jauch A. Gene expression profiling for molecular classification of multiple myeloma in newly diagnosed patients. Blood (2010) 116:2543–53. 10.1182/blood-2009-12-261032 20574050

[20] Shi L Campbell G Jones WD Campagne F Wen Z Walker SJ. The MicroArray Quality Control (MAQC)-II study of common practices for the development and validation of microarray-based predictive models. Nat Biotechnol. (2010) 28:827–38. 10.1038/nbt.1665 20676074 PMC3315840

[21] Chng WJ Kumar S Vanwier S Ahmann G Price-Troska T Henderson K. Molecular dissection of hyperdiploid multiple myeloma by gene expression profiling. Cancer Res. (2007) 67:2982–9. 10.1158/0008-5472.CAN-06-4046 17409404

[22] Tiedemann RE Zhu YX Schmidt J Yin H Shi CX Que Q. Kinome-wide RNAi studies in human multiple myeloma identify vulnerable kinase targets, including a lymphoid-restricted kinase, GRK6. Blood (2010) 115:1594–604. 10.1182/blood-2009-09-243980 19996089 PMC2830764

[23] Kryukov F Nemec P Radova L Kryukova E Okubote S Minarik J. Centrosome associated genes pattern for risk sub-stratification in multiple myeloma. J Transl Med. (2016) 14:150. 10.1186/s12967-016-0906-9 27234807 PMC4884414

[24] Gautier L Cope L Bolstad BM Irizarry RA. affy–analysis of Affymetrix GeneChip data at the probe level. Bioinformatics (2004) 20:307–15. 10.1093/bioinformatics/btg405 14960456

[25] Carvalho BS Irizarry RA. A framework for oligonucleotide microarray preprocessing. Bioinformatics (2010) 26:2363–7. 10.1093/bioinformatics/btq431 20688976 PMC2944196

[26] Irizarry RA Hobbs B Collin F Beazer-Barclay YD Antonellis KJ Scherf U. Exploration, normalization, and summaries of high density oligonucleotide array probe level data. Biostatistics (2003) 4:249–64. 10.1093/biostatistics/4.2.249 12925520

[27] Langfelder P Horvath S. WGCNA: an R package for weighted correlation network analysis. BMC Bioinformatics (2008) 9:559. 10.1186/1471-2105-9-559 19114008 PMC2631488

[28] Oldham MC Langfelder P Horvath S. Network methods for describing sample relationships in genomic datasets: application to Huntington's disease. BMC Syst Biol. (2012) 6:63. 10.1186/1752-0509-6-63 22691535 PMC3441531

[29] Li A Horvath S. Network module detection: Affinity search technique with the multi-node topological overlap measure. BMC Res Notes (2009) 2:142. 10.1186/1756-0500-2-142 19619323 PMC2727520

[30] Dong J Horvath S. Understanding network concepts in modules. BMC Syst Biol. (2007) 1:24. 10.1186/1752-0509-1-24 17547772 PMC3238286

[31] Huang da W Sherman BT Lempicki RA. Systematic and integrative analysis of large gene lists using DAVID bioinformatics resources. Nat Protoc. (2009) 4:44–57. 10.1038/nprot.2008.211 19131956

[32] Ito K Murphy D. Application of ggplot2 to Pharmacometric Graphics. CPT Pharmacometr Syst Pharmacol. (2013) 2:e79. 10.1038/psp.2013.56 24132163 PMC3817376

[33] Wang H Lengerich BJ Aragam B Xing EP. Precision lasso: accounting for correlations and linear dependencies in high-dimensional genomic data. Bioinformatics (2018). [Epub ahead of print]. 10.1093/bioinformatics/bty750 30184048 PMC6449749

[34] Friedman J Hastie T Tibshirani R. Regularization paths for generalized linear models via coordinate descent. J Stat Softw. (2010) 33:1–22. 10.18637/jss.v033.i01 20808728 PMC2929880

[35] Holleczek B Brenner H. Model based period analysis of absolute and relative survival with R: data preparation, model fitting and derivation of survival estimates. Comput Methods Programs Biomed. (2013) 110:192–202. 10.1016/j.cmpb.2012.10.004 23116692

[36] Lorent M Giral M Foucher Y. Net time-dependent ROC curves: a solution for evaluating the accuracy of a marker to predict disease-related mortality. Stat Med. (2014) 33:2379–89. 10.1002/sim.6079 24399671

[37] Li Z Xia Y Feng LN Chen JR Li HM Cui J. Genetic risk of extranodal natural killer T-cell lymphoma: a genome-wide association study. Lancet Oncol. (2016) 17:1240–7. 10.1016/S1470-2045(16)30148-6 27470079 PMC6790270

[38] Liu LM Xiong DD Lin P Yang H Dang YW Chen G. DNA topoisomerase 1 and 2A function as oncogenes in liver cancer and may be direct targets of nitidine chloride. Int J Oncol. (2018) 53:1897–912. 10.3892/ijo.2018.4531 30132517 PMC6192772

[39] Zhang R Xu J Zhao J Bai JH. Proliferation and invasion of colon cancer cells are suppressed by knockdown of TOP2A. J Cell Biochem. (2018) 119:7256–63. 10.1002/jcb.26916 29761838

[40] Kawaguchi K Senga S Kubota C Kawamura Y Ke Y Fujii H. High expression of Fatty Acid-Binding Protein 5 promotes cell growth and metastatic potential of colorectal cancer cells. FEBS Open Bio (2016) 6:190–9. 10.1002/2211-5463.12031 27047747 PMC4794781

[41] Gu CY Qin XJ Qu YY Zhu Y Wan FN Zhang GM. Genetic variants of the CYP1B1 gene as predictors of biochemical recurrence after radical prostatectomy in localized prostate cancer patients. Medicine (2016) 95:e4066. 10.1097/MD.0000000000004066 27399092 PMC5058821

[42] Ruminy P Jardin F Picquenot JM Parmentier F Contentin N Buchonnet G. S(mu) mutation patterns suggest different progression pathways in follicular lymphoma: early direct or late from FL progenitor cells. Blood (2008) 112:1951–9. 10.1182/blood-2007-11-124560 18515657

[43] Chen J Rajasekaran M Xia H Zhang X Kong SN Sekar K. The microtubule-associated protein PRC1 promotes early recurrence of hepatocellular carcinoma in association with the Wnt/beta-catenin signalling pathway. Gut (2016) 65:1522–34. 10.1136/gutjnl-2015-310625 26941395 PMC5036256

[44] Mariano FV Gomez CA de Souza doNascimento J Dos Santos HT Egal ES Montalli VA. Lysozyme expression can be useful to distinguish mammary analog secretory carcinoma from acinic cell carcinoma of salivary glands. Head Neck Pathol. (2016) 10:429–36. 10.1007/s12105-016-0718-5 27177644 PMC5082043

[45] Wu J Wang J. HMGN5 expression in bladder cancer tissue and its role on prognosis. Eur Rev Med Pharmacol Sci. (2018) 22:970–5. 10.26355/eurrev_201802_14378 29509244

