# Supplementary figures and images for: Development and Validation of a 9-Gene Prognostic Signature in Patients With Multiple Myeloma
Source: Front Oncol. 2019 Jan 8;8:615. doi: 10.3389/fonc.2018.00615 (PMC6331463; doi:10.3389/fonc.2018.00615)

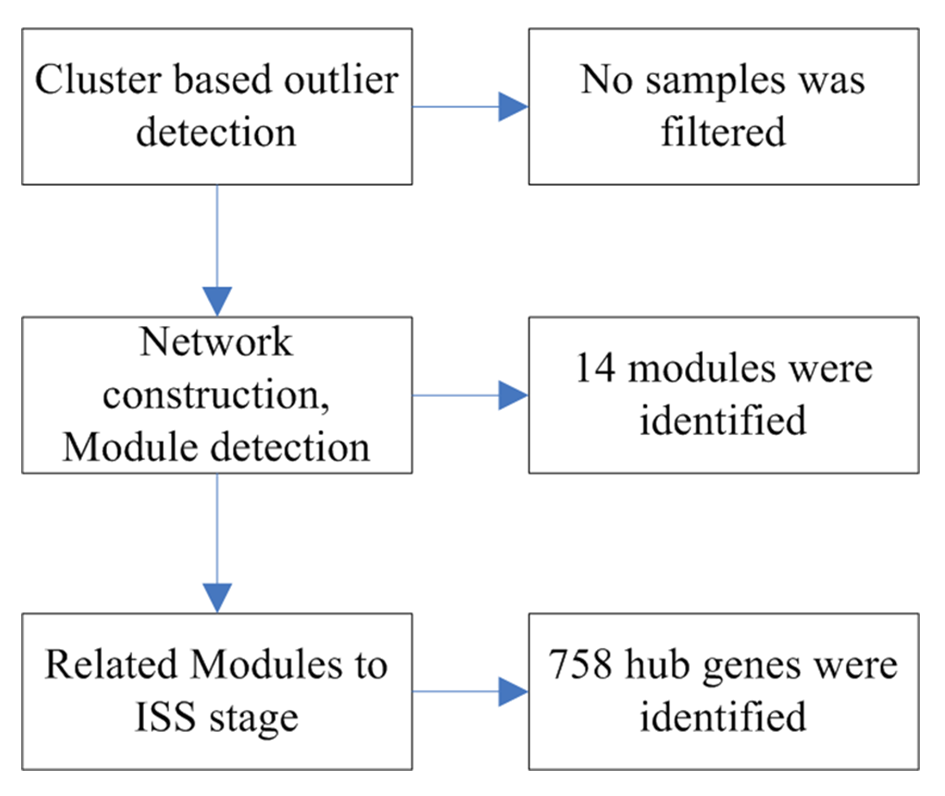

Supplement: Supplementary Figure 1 — Flowchart depicting the major process of Co-expression network construction and identification of hub genes. [file Image_1.TIF]

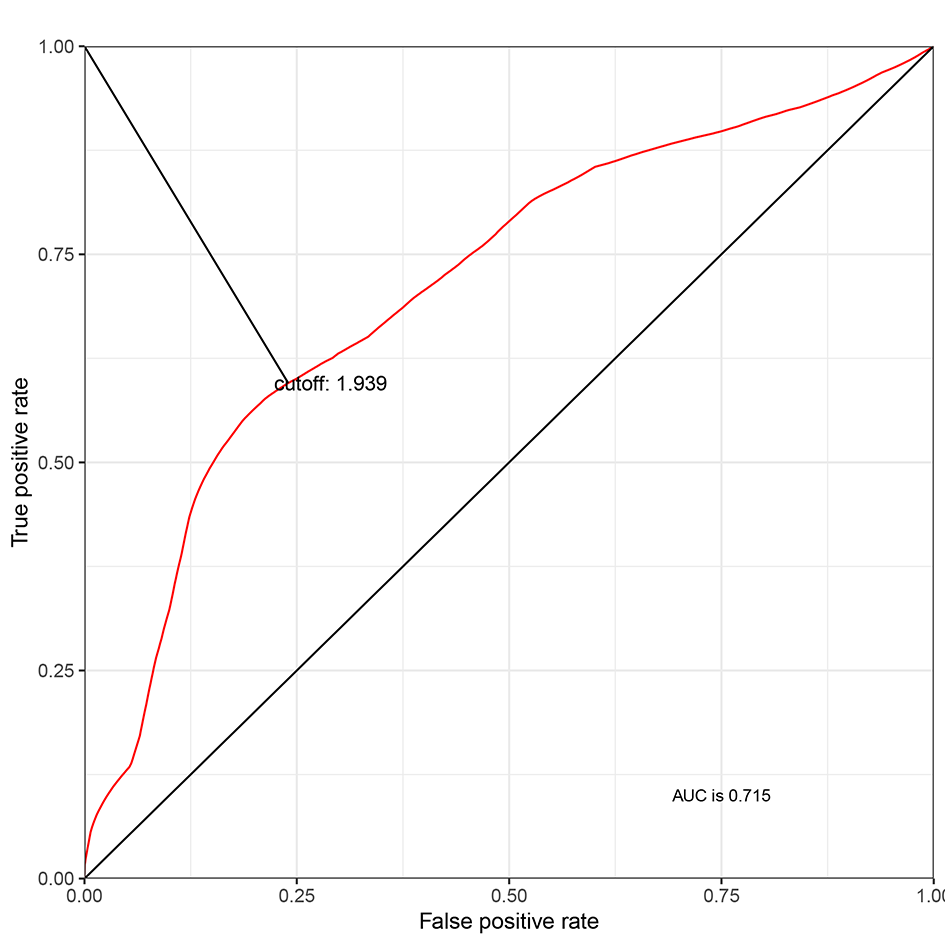

Supplement: Supplementary Figure 2 — Time-Dependent ROC Curve for the 9-gene signature in the training set at 90 months. [file Image_2.TIF]
