# Supplementary material for: Development and Validation of a 9-Gene Prognostic Signature in Patients With Multiple Myeloma
Source: Front Oncol. 2019 Jan 8;8:615. doi: 10.3389/fonc.2018.00615 (PMC6331463; doi:10.3389/fonc.2018.00615)
Supplement: Supplementary Table 1 — Hub genes calculated using weighted co- expression network analysis and their impact on the overall survival of patients with multiple myeloma. [file Table_1.docx]

**Supplementary table 1** Hub genes calculated using weighted co- expression network analysis and their impact on the overall survival of patients with multiple myeloma

| Probe Set ID | Gene Symbol | coefficients | HR | LCI | UCL | P value |
| --- | --- | --- | --- | --- | --- | --- |
| 225834_at | FAM72A | 0.454072 | 1.574712 | 1.342926 | 1.846503 | 2.28E-08 |
| 222606_at | ZWILCH | 0.787611 | 2.198139 | 1.644446 | 2.938265 | 1.04E-07 |
| 204092_s_at | AURKA | 0.557559 | 1.746404 | 1.414012 | 2.156932 | 2.27E-07 |
| 202705_at | CCNB2 | 0.382419 | 1.465827 | 1.256322 | 1.710268 | 1.18E-06 |
| 213599_at | OIP5 | 0.426248 | 1.5315 | 1.282717 | 1.828534 | 2.44E-06 |
| 221606_s_at | HMGN5 | 0.363967 | 1.439026 | 1.233895 | 1.67826 | 3.51E-06 |
| 226936_at | CENPW | 0.530686 | 1.700098 | 1.358212 | 2.128044 | 3.61E-06 |
| 208873_s_at | REEP5 | -0.77852 | 0.459083 | 0.330208 | 0.638257 | 3.64E-06 |
| 218755_at | KIF20A | 0.42333 | 1.527039 | 1.275071 | 1.828798 | 4.2E-06 |
| 1554572_a_at | SUV39H2 | 0.791504 | 2.206714 | 1.572427 | 3.096859 | 4.7E-06 |
| 202954_at | UBE2C | 0.496843 | 1.643525 | 1.327836 | 2.034268 | 4.98E-06 |
| 208079_s_at | AURKA | 0.353128 | 1.423514 | 1.222884 | 1.65706 | 5.22E-06 |
| 210052_s_at | TPX2 | 0.434242 | 1.543793 | 1.279878 | 1.862128 | 5.63E-06 |
| 212282_at | TMEM97 | 0.550928 | 1.734863 | 1.361581 | 2.210481 | 8.32E-06 |
| 214710_s_at | CCNB1 | 0.486785 | 1.627076 | 1.311691 | 2.018294 | 9.51E-06 |
| 1554768_a_at | MAD2L1 | 0.488772 | 1.630313 | 1.312378 | 2.025271 | 1.01E-05 |
| 223229_at | UBE2T | 0.4366 | 1.547438 | 1.274608 | 1.878667 | 1.03E-05 |
| 236641_at | KIF14 | 0.41293 | 1.511239 | 1.257806 | 1.815735 | 1.04E-05 |
| 202345_s_at | FABP5 | 0.3075 | 1.36002 | 1.185959 | 1.559629 | 1.08E-05 |
| 204023_at | RFC4 | 0.581173 | 1.788135 | 1.379401 | 2.317982 | 1.14E-05 |
| 203755_at | BUB1B | 0.41973 | 1.52155 | 1.260995 | 1.835942 | 1.19E-05 |
| 223381_at | NUF2 | 0.311645 | 1.365669 | 1.187711 | 1.570291 | 1.21E-05 |
| 213226_at | CCNA2 | 0.566183 | 1.76153 | 1.366315 | 2.271065 | 1.26E-05 |
| 218039_at | NUSAP1 | 0.422512 | 1.52579 | 1.259731 | 1.84804 | 1.55E-05 |
| 222680_s_at | DTL | 0.412783 | 1.511017 | 1.251549 | 1.824276 | 1.75E-05 |
| 218663_at | NCAPG | 0.330543 | 1.391724 | 1.196026 | 1.619443 | 1.91E-05 |
| 222958_s_at | DEPDC1 | 0.345503 | 1.4127 | 1.204165 | 1.65735 | 2.24E-05 |
| 204641_at | NEK2 | 0.316973 | 1.372966 | 1.185484 | 1.590099 | 2.32E-05 |
| 218350_s_at | GMNN | 0.476294 | 1.610097 | 1.291206 | 2.007745 | 2.34E-05 |
| 212021_s_at | MKI67 | 0.539223 | 1.714674 | 1.335134 | 2.202105 | 2.4E-05 |
| 230165_at | SGOL2 | 0.57753 | 1.781633 | 1.362462 | 2.329765 | 2.44E-05 |
| 209773_s_at | RRM2 | 0.330639 | 1.391857 | 1.193491 | 1.623193 | 2.5E-05 |
| 201663_s_at | SMC4 | 0.548386 | 1.730458 | 1.340333 | 2.234136 | 2.59E-05 |
| 203362_s_at | MAD2L1 | 0.394344 | 1.48341 | 1.233963 | 1.783283 | 2.69E-05 |
| 205394_at | CHEK1 | 0.497054 | 1.643871 | 1.303281 | 2.07347 | 2.72E-05 |
| 220085_at | HELLS | 0.267027 | 1.306076 | 1.151961 | 1.480809 | 3.07E-05 |
| 218073_s_at | NDC1 | 0.67252 | 1.959169 | 1.427951 | 2.688008 | 3.08E-05 |
| 202107_s_at | MCM2 | 0.463394 | 1.58946 | 1.276871 | 1.978574 | 3.36E-05 |
| 1554696_s_at | TYMS | 0.335468 | 1.398595 | 1.191235 | 1.64205 | 4.18E-05 |
| 222077_s_at | RACGAP1 | 0.441889 | 1.555643 | 1.25844 | 1.923036 | 4.41E-05 |
| 218585_s_at | DTL | 0.326521 | 1.386138 | 1.182652 | 1.624636 | 5.55E-05 |
| 213008_at | FANCI | 0.2241 | 1.251196 | 1.121988 | 1.395284 | 5.59E-05 |
| 219258_at | TIPIN | 0.562324 | 1.754747 | 1.3339 | 2.30837 | 5.84E-05 |
| 201930_at | MCM6 | 0.478859 | 1.614232 | 1.27713 | 2.040313 | 6.16E-05 |
| 228273_at | PRR11 | 0.330673 | 1.391905 | 1.183369 | 1.63719 | 6.52E-05 |
| 228323_at | CASC5 | 0.500814 | 1.650063 | 1.290198 | 2.110304 | 6.61E-05 |
| 201291_s_at | TOP2A | 0.221969 | 1.248533 | 1.118424 | 1.393778 | 7.71E-05 |
| 226980_at | DEPDC1B | 0.319115 | 1.37591 | 1.174327 | 1.612095 | 7.88E-05 |
| 202095_s_at | BIRC5 | 0.249501 | 1.283384 | 1.132969 | 1.45377 | 8.75E-05 |
| 222036_s_at | MCM4 | 0.546313 | 1.726874 | 1.314153 | 2.269213 | 8.84E-05 |
| 204444_at | KIF11 | 0.37357 | 1.452912 | 1.203646 | 1.7538 | 0.0001 |
| 204146_at | RAD51AP1 | 0.373419 | 1.452693 | 1.202775 | 1.754539 | 0.000106 |
| 218009_s_at | PRC1 | 0.374513 | 1.454283 | 1.203291 | 1.75763 | 0.000107 |
| 221685_s_at | SPDL1 | 0.633926 | 1.884996 | 1.366814 | 2.59963 | 0.000111 |
| 225827_at | AGO2 | 0.5395 | 1.71515 | 1.304244 | 2.255512 | 0.000113 |
| 201137_s_at | HLA-DPB1 | -0.25572 | 0.774361 | 0.679483 | 0.882485 | 0.000126 |
| 210559_s_at | CDK1 | 0.316598 | 1.37245 | 1.167347 | 1.61359 | 0.000126 |
| 202437_s_at | CYP1B1 | -0.21802 | 0.804113 | 0.719001 | 0.8993 | 0.000134 |
| 212762_s_at | TCF7L2 | -0.43219 | 0.649084 | 0.519793 | 0.810534 | 0.000137 |
| 218542_at | CEP55 | 0.319198 | 1.376024 | 1.167498 | 1.621794 | 0.000141 |
| 203764_at | DLGAP5 | 0.430543 | 1.538093 | 1.229456 | 1.924209 | 0.000165 |
| 209714_s_at | CDKN3 | 0.466076 | 1.593728 | 1.250484 | 2.031189 | 0.000166 |
| 204620_s_at | VCAN | -0.46821 | 0.626121 | 0.490709 | 0.798901 | 0.000166 |
| 207165_at | HMMR | 0.358121 | 1.430639 | 1.186743 | 1.724661 | 0.000173 |
| 235113_at | LRR1 | 0.626157 | 1.870409 | 1.348876 | 2.593588 | 0.000174 |
| 204026_s_at | ZWINT | 0.402244 | 1.495176 | 1.211122 | 1.845852 | 0.000183 |
| 209421_at | MSH2 | 0.424939 | 1.529497 | 1.223486 | 1.912044 | 0.000191 |
| 204033_at | TRIP13 | 0.356273 | 1.427997 | 1.183511 | 1.722987 | 0.0002 |
| 219990_at | E2F8 | 0.369524 | 1.447046 | 1.190662 | 1.758637 | 0.000204 |
| 232065_x_at | CENPL | 0.64123 | 1.898815 | 1.35355 | 2.663735 | 0.000205 |
| 202236_s_at | SLC16A1 | 0.681354 | 1.976552 | 1.378902 | 2.83324 | 0.000208 |
| 209642_at | BUB1 | 0.354898 | 1.426035 | 1.182152 | 1.720233 | 0.000208 |
| 201897_s_at | CKS1B | 0.390384 | 1.477548 | 1.202008 | 1.81625 | 0.00021 |
| 228729_at | CCNB1 | 0.28199 | 1.325766 | 1.142088 | 1.538984 | 0.000211 |
| 201890_at | RRM2 | 0.274479 | 1.315845 | 1.137956 | 1.521541 | 0.000212 |
| 202503_s_at | KIAA0101 | 0.348895 | 1.4175 | 1.178034 | 1.705643 | 0.00022 |
| 208103_s_at | ANP32E | 0.542947 | 1.721072 | 1.289066 | 2.297857 | 0.000232 |
| 228069_at | MTFR2 | 0.43438 | 1.544005 | 1.224342 | 1.947128 | 0.000242 |
| 206102_at | GINS1 | 0.329059 | 1.38966 | 1.165404 | 1.657069 | 0.000248 |
| 203968_s_at | CDC6 | 0.445682 | 1.561554 | 1.230134 | 1.982265 | 0.000251 |
| 203432_at | TMPO | 0.507845 | 1.661707 | 1.264385 | 2.183883 | 0.00027 |
| 1555758_a_at | CDKN3 | 0.29887 | 1.348334 | 1.147528 | 1.58428 | 0.000281 |
| 204822_at | TTK | 0.34732 | 1.415269 | 1.173165 | 1.707337 | 0.000285 |
| 203214_x_at | CDK1 | 0.351905 | 1.421774 | 1.175628 | 1.719457 | 0.000285 |
| 216237_s_at | MCM5 | 0.468979 | 1.598361 | 1.239964 | 2.060349 | 0.000294 |
| 211654_x_at | HLA-DQB1 | -0.46294 | 0.629428 | 0.48986 | 0.80876 | 0.000295 |
| 201292_at | TOP2A | 0.263681 | 1.301713 | 1.127023 | 1.503481 | 0.000335 |
| 219493_at | SHCBP1 | 0.265982 | 1.304711 | 1.127819 | 1.509348 | 0.000346 |
| 219918_s_at | ASPM | 0.232318 | 1.261521 | 1.110435 | 1.433163 | 0.000358 |
| 226287_at | CCDC34 | 0.450524 | 1.569135 | 1.224119 | 2.011393 | 0.000376 |
| 201990_s_at | CREBL2 | -0.63903 | 0.527804 | 0.371109 | 0.750662 | 0.000377 |
| 220651_s_at | MCM10 | 0.218033 | 1.243628 | 1.102138 | 1.403283 | 0.000403 |
| 209709_s_at | HMMR | 0.458688 | 1.581997 | 1.226261 | 2.040932 | 0.000416 |
| 203358_s_at | EZH2 | 0.396201 | 1.486168 | 1.192403 | 1.852305 | 0.000422 |
| 223307_at | CDCA3 | 0.52442 | 1.689479 | 1.261568 | 2.262534 | 0.000433 |
| 212543_at | AIM1 | -0.38125 | 0.683004 | 0.552243 | 0.844726 | 0.000438 |
| 221731_x_at | VCAN | -0.36828 | 0.691925 | 0.56273 | 0.850782 | 0.000479 |
| 202870_s_at | CDC20 | 0.293394 | 1.340971 | 1.13671 | 1.581935 | 0.000502 |
| 235545_at | DEPDC1 | 0.33965 | 1.404456 | 1.158578 | 1.702516 | 0.000542 |
| 204709_s_at | KIF23 | 0.216279 | 1.241449 | 1.097516 | 1.404258 | 0.000582 |
| 225687_at | FAM83D | 0.507192 | 1.660622 | 1.23842 | 2.226761 | 0.000702 |
| 203209_at | RFC5 | 0.620501 | 1.859859 | 1.298477 | 2.66395 | 0.000713 |
| 218979_at | RMI1 | 0.666778 | 1.947951 | 1.323914 | 2.866132 | 0.000714 |
| 231175_at | BEND6 | -0.20533 | 0.814377 | 0.722646 | 0.917753 | 0.000758 |
| 228597_at | MIS18A | 0.46651 | 1.59442 | 1.21496 | 2.092392 | 0.000768 |
| 220865_s_at | PDSS1 | 0.669426 | 1.953116 | 1.322315 | 2.884835 | 0.000769 |
| 221059_s_at | COTL1 | -0.33381 | 0.716192 | 0.588791 | 0.871159 | 0.000837 |
| 213253_at | SMC2 | 0.440851 | 1.554029 | 1.199752 | 2.012921 | 0.000839 |
| 213975_s_at | LYZ | -0.1567 | 0.854962 | 0.779413 | 0.937834 | 0.000901 |
| 203213_at | CDK1 | 0.24626 | 1.279232 | 1.105302 | 1.480533 | 0.000958 |
| 227031_at | SNX13 | -0.54957 | 0.577198 | 0.416513 | 0.799872 | 0.000962 |
| 203967_at | CDC6 | 0.364419 | 1.439678 | 1.159319 | 1.787836 | 0.000975 |
| 212761_at | TCF7L2 | -0.42169 | 0.655937 | 0.510391 | 0.842986 | 0.000987 |
| 204276_at | TK2 | -0.52079 | 0.594051 | 0.43559 | 0.810157 | 0.001002 |
| 228401_at | ATAD2 | 0.374777 | 1.454666 | 1.163514 | 1.818675 | 0.001005 |
| 219148_at | PBK | 0.292081 | 1.339212 | 1.124076 | 1.595523 | 0.001079 |
| 217362_x_at | HLA-DRB6 | -0.30562 | 0.736664 | 0.612997 | 0.88528 | 0.001116 |
| 226150_at | PPAPDC1B | -0.41341 | 0.661392 | 0.515765 | 0.848135 | 0.001122 |
| 202589_at | TYMS | 0.282285 | 1.326156 | 1.118913 | 1.571784 | 0.00113 |
| 209374_s_at | IGHM | -0.12085 | 0.886167 | 0.823356 | 0.953769 | 0.001273 |
| 244313_at | CR1 | -0.31354 | 0.730858 | 0.603695 | 0.884807 | 0.001305 |
| 231772_x_at | CENPH | 0.367632 | 1.444311 | 1.153585 | 1.808305 | 0.001347 |
| 226826_at | LSM11 | 0.437752 | 1.54922 | 1.184762 | 2.025792 | 0.001379 |
| 204256_at | ELOVL6 | 0.302102 | 1.3527 | 1.123568 | 1.628559 | 0.001421 |
| 210983_s_at | MCM7 | 0.353775 | 1.424434 | 1.145499 | 1.771291 | 0.001464 |
| 204962_s_at | CENPA | 0.251909 | 1.286478 | 1.101459 | 1.502576 | 0.001474 |
| 218355_at | KIF4A | 0.268086 | 1.307459 | 1.108273 | 1.542445 | 0.001478 |
| 235088_at | C4orf46 | 0.486678 | 1.626902 | 1.204674 | 2.197118 | 0.0015 |
| 226450_at | INSR | -0.47202 | 0.623743 | 0.465716 | 0.835392 | 0.001543 |
| 227212_s_at | PHF19 | 0.18761 | 1.206363 | 1.073803 | 1.355288 | 0.001584 |
| 200799_at | HSPA1A | -0.2122 | 0.808803 | 0.708703 | 0.923042 | 0.001644 |
| 232238_at | ASPM | 0.242042 | 1.273847 | 1.095584 | 1.481115 | 0.001651 |
| 235141_at | MARVELD2 | -0.34068 | 0.711284 | 0.574861 | 0.880083 | 0.001715 |
| 206028_s_at | MERTK | -0.31694 | 0.728378 | 0.596576 | 0.8893 | 0.001859 |
| 206488_s_at | CD36 | -0.28106 | 0.754985 | 0.631645 | 0.902411 | 0.002013 |
| 45633_at | GINS3 | 0.241133 | 1.27269 | 1.091942 | 1.483357 | 0.002033 |
| 226661_at | CDCA2 | 0.295249 | 1.343461 | 1.113581 | 1.620796 | 0.002046 |
| 209555_s_at | CD36 | -0.1869 | 0.829531 | 0.736544 | 0.934256 | 0.002063 |
| 222608_s_at | ANLN | 0.315833 | 1.371401 | 1.120179 | 1.678963 | 0.002219 |
| 204122_at | TYROBP | -0.29209 | 0.7467 | 0.619113 | 0.900581 | 0.002248 |
| 204411_at | KIF21B | 0.166911 | 1.181649 | 1.061626 | 1.315241 | 0.002256 |
| 211991_s_at | HLA-DPA1 | -0.17962 | 0.835585 | 0.744542 | 0.937761 | 0.002275 |
| 202435_s_at | CYP1B1 | -0.30793 | 0.734963 | 0.603054 | 0.895726 | 0.00228 |
| 213007_at | FANCI | 0.322089 | 1.380008 | 1.121632 | 1.697904 | 0.002325 |
| 204127_at | RFC3 | 0.404838 | 1.499059 | 1.154912 | 1.945757 | 0.002348 |
| 212533_at | WEE1 | 0.245527 | 1.278295 | 1.090983 | 1.497766 | 0.002389 |
| 209053_s_at | WHSC1 | 0.113739 | 1.12046 | 1.040824 | 1.206189 | 0.002497 |
| 209687_at | CXCL12 | -0.16914 | 0.844387 | 0.756484 | 0.942503 | 0.002563 |
| 226051_at | SELM | -0.24457 | 0.783038 | 0.66785 | 0.918094 | 0.00259 |
| 219787_s_at | ECT2 | 0.383404 | 1.467271 | 1.143318 | 1.883016 | 0.002593 |
| 214038_at | CCL8 | -0.27148 | 0.762254 | 0.63878 | 0.909596 | 0.002605 |
| 205393_s_at | CHEK1 | 0.359915 | 1.433208 | 1.132985 | 1.812984 | 0.002691 |
| 219000_s_at | DSCC1 | 0.26662 | 1.305544 | 1.095445 | 1.55594 | 0.002899 |
| 203418_at | CCNA2 | 0.354008 | 1.424767 | 1.128548 | 1.798738 | 0.002912 |
| 206150_at | CD27 | -0.19636 | 0.821719 | 0.721974 | 0.935243 | 0.00294 |
| 204809_at | CLPX | 0.690085 | 1.993886 | 1.264733 | 3.143416 | 0.002967 |
| 219555_s_at | CENPN | 0.229199 | 1.257592 | 1.080624 | 1.463542 | 0.003056 |
| 204162_at | NDC80 | 0.229814 | 1.258366 | 1.080806 | 1.465097 | 0.003064 |
| 212827_at | IGHM | -0.25516 | 0.774795 | 0.653642 | 0.918403 | 0.003271 |
| 228766_at | CD36 | -0.23405 | 0.791325 | 0.676902 | 0.925091 | 0.003313 |
| 218746_at | TAPBPL | -0.37765 | 0.685468 | 0.532457 | 0.882451 | 0.003387 |
| 222843_at | FIGNL1 | 0.414614 | 1.513787 | 1.146072 | 1.999481 | 0.003497 |
| 212342_at | YIPF6 | -0.48242 | 0.617285 | 0.446476 | 0.853442 | 0.003514 |
| 218747_s_at | TAPBPL | -0.36926 | 0.691245 | 0.539304 | 0.885994 | 0.003548 |
| 201202_at | PCNA | 0.40904 | 1.505372 | 1.143338 | 1.982041 | 0.003564 |
| 203227_s_at | TSPAN31 | -0.63629 | 0.529253 | 0.344176 | 0.813852 | 0.003754 |
| 227350_at | HELLS | 0.321875 | 1.379712 | 1.109601 | 1.715577 | 0.003785 |
| 210054_at | HAUS3 | 0.609451 | 1.839421 | 1.212042 | 2.791546 | 0.00419 |
| 202436_s_at | CYP1B1 | -0.39252 | 0.675355 | 0.516234 | 0.883523 | 0.004192 |
| 223274_at | TCF19 | 0.410341 | 1.507332 | 1.136105 | 1.999859 | 0.004448 |
| 205909_at | POLE2 | 0.231745 | 1.260798 | 1.074687 | 1.47914 | 0.004457 |
| 208306_x_at | HLA-DRB1 | -0.25933 | 0.771567 | 0.645281 | 0.922568 | 0.004459 |
| 238762_at | MTHFD2L | 0.425699 | 1.53066 | 1.141271 | 2.052903 | 0.00448 |
| 218831_s_at | FCGRT | -0.22614 | 0.797603 | 0.682329 | 0.932351 | 0.004519 |
| 204254_s_at | VDR | -0.41174 | 0.662497 | 0.498463 | 0.88051 | 0.004559 |
| 203932_at | HLA-DMB | -0.47704 | 0.620621 | 0.44606 | 0.863493 | 0.004641 |
| 213056_at | FRMD4B | -0.20447 | 0.81508 | 0.707268 | 0.939326 | 0.004733 |
| 223454_at | CXCL16 | -0.28311 | 0.753434 | 0.618823 | 0.917326 | 0.004813 |
| 202635_s_at | POLR2K | 0.609482 | 1.839478 | 1.203685 | 2.811099 | 0.004851 |
| 223044_at | SLC40A1 | -0.18268 | 0.833036 | 0.733512 | 0.946062 | 0.004891 |
| 203666_at | CXCL12 | -0.30278 | 0.738765 | 0.597762 | 0.913027 | 0.005078 |
| 214181_x_at | LST1 | -0.1943 | 0.823408 | 0.718732 | 0.94333 | 0.005095 |
| 1560916_a_at | DPY19L1 | 0.238416 | 1.269237 | 1.07254 | 1.502007 | 0.005519 |
| 223320_s_at | ABCB10 | 0.458277 | 1.581348 | 1.143355 | 2.187126 | 0.005613 |
| 200878_at | EPAS1 | -0.1887 | 0.828033 | 0.724414 | 0.946473 | 0.005666 |
| 210986_s_at | TPM1 | -0.24318 | 0.784133 | 0.659382 | 0.932485 | 0.005948 |
| 228071_at | GIMAP7 | -0.20013 | 0.818628 | 0.70971 | 0.944261 | 0.006009 |
| 219588_s_at | NCAPG2 | 0.283104 | 1.327243 | 1.084359 | 1.62453 | 0.006045 |
| 219306_at | KIF15 | 0.296324 | 1.344905 | 1.087608 | 1.663072 | 0.006236 |
| 223296_at | SLC25A33 | 0.513873 | 1.671753 | 1.155784 | 2.418065 | 0.006357 |
| 201743_at | CD14 | -0.17805 | 0.836898 | 0.736415 | 0.951092 | 0.006365 |
| 1552316_a_at | GIMAP1 | -0.1853 | 0.830858 | 0.727064 | 0.94947 | 0.006498 |
| 222740_at | ATAD2 | 0.23165 | 1.260678 | 1.066873 | 1.48969 | 0.006527 |
| 223452_s_at | ATL3 | 0.322927 | 1.381164 | 1.094417 | 1.743042 | 0.006531 |
| 204670_x_at | HLA-DRB1 | -0.21377 | 0.807533 | 0.691891 | 0.942504 | 0.00671 |
| 209891_at | SPC25 | 0.260884 | 1.298077 | 1.073589 | 1.569504 | 0.007083 |
| 205542_at | STEAP1 | 0.281442 | 1.325039 | 1.0791 | 1.62703 | 0.007216 |
| 226218_at | IL7R | -0.20962 | 0.810891 | 0.695856 | 0.944944 | 0.007243 |
| 212374_at | FEM1B | 0.308181 | 1.360948 | 1.086111 | 1.705331 | 0.007413 |
| 202803_s_at | ITGB2 | -0.15792 | 0.853917 | 0.760572 | 0.958718 | 0.007501 |
| 209628_at | NXT2 | 0.350174 | 1.419314 | 1.096503 | 1.837161 | 0.007821 |
| 211990_at | HLA-DPA1 | -0.18431 | 0.831676 | 0.72539 | 0.953535 | 0.008243 |
| 239214_at | LOC100130458 | -0.20676 | 0.813219 | 0.696453 | 0.949561 | 0.008938 |
| 204232_at | FCER1G | -0.31085 | 0.732827 | 0.580487 | 0.925145 | 0.00894 |
| 215193_x_at | HLA-DQB1 | -0.2411 | 0.785764 | 0.655297 | 0.942207 | 0.009253 |
| 220088_at | C5AR1 | -0.14267 | 0.867042 | 0.778558 | 0.965584 | 0.009387 |
| 205237_at | FCN1 | -0.20437 | 0.815161 | 0.698665 | 0.951081 | 0.009393 |
| 225730_s_at | THUMPD3 | 0.651554 | 1.91852 | 1.17312 | 3.137546 | 0.009427 |
| 205339_at | STIL | 0.507653 | 1.661387 | 1.132234 | 2.437841 | 0.009466 |
| 203535_at | S100A9 | -0.12254 | 0.884671 | 0.806421 | 0.970513 | 0.009503 |
| 217894_at | KCTD3 | 0.33385 | 1.396334 | 1.084456 | 1.797905 | 0.009636 |
| 207039_at | CDKN2A | 0.26963 | 1.309479 | 1.067353 | 1.606531 | 0.009742 |
| 204007_at | FCGR3B | -0.31556 | 0.729379 | 0.574095 | 0.926664 | 0.00978 |
| 201477_s_at | RRM1 | 0.447352 | 1.564165 | 1.113395 | 2.197434 | 0.009901 |
| 202534_x_at | DHFR | 0.377256 | 1.458278 | 1.093239 | 1.945205 | 0.010276 |
| 202917_s_at | S100A8 | -0.14479 | 0.865204 | 0.77462 | 0.966381 | 0.010287 |
| 203868_s_at | VCAM1 | -0.16938 | 0.84419 | 0.741362 | 0.96128 | 0.010593 |
| 235609_at | BRIP1 | 0.252453 | 1.287179 | 1.060262 | 1.562662 | 0.010731 |
| 215176_x_at | IGK | -0.12496 | 0.882531 | 0.801574 | 0.971665 | 0.010913 |
| 203645_s_at | CD163 | -0.13588 | 0.872947 | 0.786151 | 0.969326 | 0.01099 |
| 219073_s_at | OSBPL10 | -0.16266 | 0.849879 | 0.748314 | 0.96523 | 0.012247 |
| 202843_at | DNAJB9 | -0.37564 | 0.686848 | 0.511708 | 0.921934 | 0.012378 |
| 228376_at | GGTA1P | -0.38993 | 0.677108 | 0.498806 | 0.919145 | 0.012396 |
| 235509_at | LOC100506538 | 0.413966 | 1.512806 | 1.092684 | 2.094459 | 0.012633 |
| 200839_s_at | CTSB | -0.2874 | 0.750209 | 0.598319 | 0.940658 | 0.012775 |
| 225274_at | PCYOX1 | -0.40835 | 0.664748 | 0.481119 | 0.918463 | 0.013301 |
| 202890_at | MAP7 | -0.09865 | 0.906061 | 0.837961 | 0.979695 | 0.013341 |
| 230550_at | MS4A6A | -0.17166 | 0.842263 | 0.735133 | 0.965005 | 0.013392 |
| 226751_at | CNRIP1 | -0.2027 | 0.816519 | 0.695338 | 0.95882 | 0.013399 |
| 222848_at | CENPK | 0.222714 | 1.249463 | 1.04707 | 1.490977 | 0.013507 |
| 205382_s_at | CFD | -0.18385 | 0.832064 | 0.719014 | 0.962889 | 0.013604 |
| 227211_at | PHF19 | 0.201568 | 1.223319 | 1.041027 | 1.437532 | 0.014351 |
| 217378_x_at | IGKV1OR2-108 | -0.09991 | 0.904918 | 0.835264 | 0.98038 | 0.014492 |
| 216207_x_at | IGKC | -0.13822 | 0.870909 | 0.779343 | 0.973234 | 0.014742 |
| 209312_x_at | HLA-DQB1 | -0.20573 | 0.814049 | 0.689793 | 0.960688 | 0.01491 |
| 203382_s_at | APOE | -0.12824 | 0.87964 | 0.793308 | 0.975368 | 0.014967 |
| 203973_s_at | CEBPD | -0.18181 | 0.83376 | 0.720142 | 0.965303 | 0.014999 |
| 227266_s_at | FYB | -0.16415 | 0.848612 | 0.743263 | 0.968892 | 0.015214 |
| 225911_at | NPNT | 0.121474 | 1.129159 | 1.023608 | 1.245595 | 0.015268 |
| 204825_at | MELK | 0.230047 | 1.258659 | 1.045075 | 1.515894 | 0.015323 |
| 208955_at | DUT | 0.48755 | 1.628323 | 1.097584 | 2.415701 | 0.015409 |
| 223343_at | MS4A7 | -0.14536 | 0.864711 | 0.768773 | 0.972622 | 0.015409 |
| 214151_s_at | CCPG1 | -0.31554 | 0.729393 | 0.564815 | 0.941926 | 0.015583 |
| 225207_at | PDK4 | -0.1401 | 0.869272 | 0.775953 | 0.973813 | 0.015609 |
| 1552386_at | GAPT | -0.18362 | 0.832253 | 0.717143 | 0.96584 | 0.015623 |
| 204959_at | MNDA | -0.18741 | 0.8291 | 0.71165 | 0.965934 | 0.016186 |
| 212998_x_at | HLA-DQB1 | -0.13451 | 0.874145 | 0.782992 | 0.975909 | 0.016666 |
| 200916_at | TAGLN2 | 0.153021 | 1.16535 | 1.028122 | 1.320894 | 0.016674 |
| 201811_x_at | SH3BP5 | -0.22197 | 0.800939 | 0.667571 | 0.960953 | 0.016914 |
| 217157_x_at | IGK | -0.14665 | 0.863595 | 0.765557 | 0.974189 | 0.017065 |
| 213915_at | NKG7 | -0.17081 | 0.842979 | 0.732268 | 0.970427 | 0.017414 |
| 208943_s_at | SEC62 | -0.42252 | 0.655393 | 0.461261 | 0.931231 | 0.018399 |
| 203543_s_at | KLF9 | -0.12848 | 0.879431 | 0.790015 | 0.978967 | 0.018847 |
| 211656_x_at | HLA-DQB1 | -0.17311 | 0.841041 | 0.727677 | 0.972066 | 0.019104 |
| 214370_at | S100A8 | -0.12965 | 0.878407 | 0.787852 | 0.979371 | 0.019519 |
| 225399_at | TSEN15 | 0.309807 | 1.363162 | 1.050987 | 1.768063 | 0.019557 |
| 204619_s_at | VCAN | -0.15324 | 0.857928 | 0.753791 | 0.976451 | 0.020292 |
| 203501_at | CPQ | -0.18641 | 0.829934 | 0.70885 | 0.971701 | 0.020518 |
| 216853_x_at | IGLJ3 | -0.12625 | 0.881396 | 0.791647 | 0.981319 | 0.021215 |
| 244519_at | ASXL1 | 0.317452 | 1.373623 | 1.047835 | 1.800705 | 0.021548 |
| 217320_at | LOC100293211 | -0.13566 | 0.873138 | 0.777617 | 0.980393 | 0.021736 |
| 223758_s_at | GTF2H2 | 0.293363 | 1.34093 | 1.043693 | 1.722819 | 0.021765 |
| 205786_s_at | ITGAM | -0.19301 | 0.824473 | 0.698851 | 0.972677 | 0.022113 |
| 228468_at | MASTL | 0.398196 | 1.489135 | 1.058699 | 2.094575 | 0.022157 |
| 226818_at | MPEG1 | -0.17351 | 0.840706 | 0.724181 | 0.97598 | 0.022646 |
| 230012_at | LINC00324 | -0.19041 | 0.826624 | 0.701612 | 0.97391 | 0.022848 |
| 234973_at | SLC38A5 | 0.268612 | 1.308147 | 1.035568 | 1.652474 | 0.024251 |
| 212330_at | TFDP1 | 0.368893 | 1.446133 | 1.049156 | 1.993317 | 0.024257 |
| 218662_s_at | NCAPG | 0.369709 | 1.447313 | 1.047924 | 1.998919 | 0.024826 |
| 223344_s_at | MS4A7 | -0.19505 | 0.82279 | 0.693626 | 0.976005 | 0.025174 |
| 202833_s_at | SERPINA1 | -0.15464 | 0.856723 | 0.747877 | 0.98141 | 0.025705 |
| 219294_at | CENPQ | 0.333165 | 1.395378 | 1.040621 | 1.871075 | 0.026014 |
| 227627_at | C8orf44-SGK3 | -0.33427 | 0.715864 | 0.532976 | 0.961509 | 0.026369 |
| 235728_at | ZFP3 | -0.15665 | 0.855005 | 0.744215 | 0.982288 | 0.026942 |
| 222777_s_at | WHSC1 | 0.141281 | 1.151748 | 1.016062 | 1.305554 | 0.027166 |
| 202094_at | BIRC5 | 0.22737 | 1.255294 | 1.025426 | 1.536692 | 0.027576 |
| 201041_s_at | DUSP1 | -0.23584 | 0.78991 | 0.64031 | 0.974464 | 0.027706 |
| 201313_at | ENO2 | -0.11157 | 0.894431 | 0.809717 | 0.988007 | 0.027976 |
| 218781_at | SMC6 | 0.418659 | 1.519922 | 1.045842 | 2.208903 | 0.028167 |
| 202561_at | TNKS | -0.31044 | 0.733122 | 0.555245 | 0.967985 | 0.028564 |
| 238513_at | PRRG4 | -0.16291 | 0.849664 | 0.734339 | 0.983101 | 0.0286 |
| 234884_x_at | CKAP2 | -0.15051 | 0.860272 | 0.75178 | 0.984422 | 0.028652 |
| 207332_s_at | TFRC | 0.28054 | 1.323844 | 1.027417 | 1.705795 | 0.030075 |
| 228771_at | ADRBK2 | -0.26694 | 0.765716 | 0.601156 | 0.975322 | 0.030589 |
| 229560_at | TLR8 | -0.20237 | 0.816797 | 0.679312 | 0.982107 | 0.031401 |
| 235463_s_at | CERS6 | 0.200733 | 1.222298 | 1.017799 | 1.467886 | 0.031649 |
| 206110_at | HIST1H3A J | 0.173479 | 1.189435 | 1.014979 | 1.393878 | 0.032058 |
| 208018_s_at | HCK | -0.21232 | 0.808708 | 0.665892 | 0.982155 | 0.032227 |
| 1553338_at | SDE2 | 0.312354 | 1.366639 | 1.025955 | 1.820453 | 0.032752 |
| 202149_at | NEDD9 | -0.22168 | 0.801174 | 0.652788 | 0.98329 | 0.033904 |
| 201798_s_at | MYOF | -0.14806 | 0.862376 | 0.751683 | 0.989369 | 0.034647 |
| 32128_at | CCL18 | -0.13143 | 0.876842 | 0.775985 | 0.990809 | 0.035025 |
| 226757_at | IFIT2 | -0.14317 | 0.866605 | 0.758493 | 0.990126 | 0.035212 |
| 219090_at | SLC24A3 | -0.16042 | 0.851786 | 0.733618 | 0.988988 | 0.035267 |
| 224616_at | DYNC1LI2 | -0.28652 | 0.750875 | 0.574072 | 0.982131 | 0.036475 |
| 209054_s_at | WHSC1 | 0.143145 | 1.153897 | 1.008373 | 1.320423 | 0.037416 |
| 238015_at | C4orf46 | 0.227846 | 1.255892 | 1.012526 | 1.557753 | 0.038151 |
| 201427_s_at | SEPP1 | -0.13479 | 0.873897 | 0.769094 | 0.992982 | 0.038639 |
| 210175_at | GCFC2 | 0.261988 | 1.299511 | 1.013501 | 1.666234 | 0.038856 |
| 211868_x_at | IGH | -0.13638 | 0.87251 | 0.766399 | 0.993314 | 0.039268 |
| 1554251_at | HP1BP3 | 0.367236 | 1.443738 | 1.016627 | 2.050291 | 0.040159 |
| 214916_x_at | IGHA1 | -0.10424 | 0.901009 | 0.815609 | 0.995349 | 0.040198 |
| 207431_s_at | DEGS1 | 0.386405 | 1.471681 | 1.017283 | 2.129048 | 0.040276 |
| 220295_x_at | DEPDC1 | 0.291814 | 1.338854 | 1.012445 | 1.770497 | 0.040687 |
| 217933_s_at | LAP3 | 0.278643 | 1.321336 | 1.011806 | 1.725556 | 0.040741 |
| 212442_s_at | CERS6 | 0.134722 | 1.144219 | 1.004367 | 1.303544 | 0.042818 |
| 1555274_a_at | EPT1 | 0.305329 | 1.357071 | 1.009377 | 1.824533 | 0.0432 |
| 223204_at | FAM198B | -0.13219 | 0.876174 | 0.770368 | 0.996511 | 0.044095 |
| 224356_x_at | MS4A6A | -0.13069 | 0.877492 | 0.772437 | 0.996836 | 0.044571 |
| 213274_s_at | CTSB | -0.31691 | 0.728393 | 0.534547 | 0.992536 | 0.044705 |
| 227936_at | TMEM68 | 0.424142 | 1.528279 | 1.008861 | 2.315121 | 0.045328 |
| 235299_at | SLC41A2 | -0.31342 | 0.730946 | 0.537456 | 0.994094 | 0.045747 |
| 238429_at | TMEM71 | -0.17952 | 0.835673 | 0.700562 | 0.996842 | 0.04603 |
| 213817_at | IRAK3 | -0.10505 | 0.900279 | 0.811833 | 0.99836 | 0.046473 |
| 206214_at | PLA2G7 | -0.13175 | 0.876556 | 0.769694 | 0.998255 | 0.047001 |
| 218145_at | TRIB3 | 0.225773 | 1.253291 | 1.002062 | 1.567506 | 0.047927 |
| 213095_x_at | AIF1 | -0.19459 | 0.823172 | 0.678623 | 0.998511 | 0.048259 |
| 216576_x_at | IGK | -0.08359 | 0.919807 | 0.846533 | 0.999424 | 0.04843 |
| 241224_x_at | DSCR8 | 0.107205 | 1.113162 | 1.000642 | 1.238334 | 0.048636 |
| 215049_x_at | CD163 | -0.09148 | 0.91258 | 0.833121 | 0.999618 | 0.049046 |
| 1552398_a_at | CLEC12A | -0.1436 | 0.866234 | 0.750788 | 0.999431 | 0.049096 |
| 209969_s_at | STAT1 | 0.121923 | 1.129667 | 1.000467 | 1.275552 | 0.049126 |
| 211429_s_at | SERPINA1 | -0.20682 | 0.813163 | 0.661692 | 0.999307 | 0.049235 |
| 208438_s_at | FGR | -0.12844 | 0.879464 | 0.773723 | 0.999657 | 0.049389 |
| 204783_at | MLF1 | 0.22795 | 1.256023 | 1.000275 | 1.57716 | 0.049724 |
| 218559_s_at | MAFB | -0.094 | 0.910283 | 0.828202 | 1.000499 | 0.051222 |
| 211645_x_at | IGKV1-17 | -0.08339 | 0.919996 | 0.845959 | 1.000512 | 0.051414 |
| 204112_s_at | HNMT | -0.12391 | 0.883463 | 0.779905 | 1.000771 | 0.051433 |
| 228565_at | KIAA1804 | 0.209794 | 1.233424 | 0.997316 | 1.525429 | 0.052969 |
| 225352_at | SEC62 | -0.33075 | 0.718383 | 0.513222 | 1.005556 | 0.053896 |
| 225655_at | UHRF1 | 0.159225 | 1.172602 | 0.997331 | 1.378674 | 0.053904 |
| 227847_at | EPM2AIP1 | -0.42215 | 0.655634 | 0.426722 | 1.007344 | 0.054033 |
| 226298_at | RUNDC1 | -0.3741 | 0.68791 | 0.469976 | 1.006901 | 0.054282 |
| 213275_x_at | CTSB | -0.26876 | 0.764329 | 0.581187 | 1.005184 | 0.054484 |
| 216401_x_at | IGKV1-37 | -0.07532 | 0.927446 | 0.858714 | 1.00168 | 0.055207 |
| 202087_s_at | CTSL | -0.16997 | 0.843693 | 0.708656 | 1.004463 | 0.056143 |
| 217145_at | IGK | -0.11071 | 0.895194 | 0.799018 | 1.002947 | 0.056233 |
| 223556_at | HELLS | 0.317038 | 1.373054 | 0.991552 | 1.901341 | 0.056277 |
| 218862_at | ASB13 | 0.156514 | 1.169427 | 0.995236 | 1.374105 | 0.057179 |
| 226932_at | SSPN | -0.24157 | 0.785398 | 0.611868 | 1.008142 | 0.057919 |
| 209480_at | HLA-DQB1 | -0.11808 | 0.888628 | 0.786025 | 1.004623 | 0.059257 |
| 228532_at | C1orf162 | -0.13804 | 0.87106 | 0.754561 | 1.005545 | 0.0595 |
| 202131_s_at | RIOK3 | 0.316245 | 1.371966 | 0.986732 | 1.9076 | 0.060035 |
| 206390_x_at | PF4 | -0.09302 | 0.911177 | 0.826625 | 1.004377 | 0.061199 |
| 207307_at | HTR2C | 0.097694 | 1.102626 | 0.995245 | 1.221592 | 0.061651 |
| 219910_at | FICD | -0.2601 | 0.770975 | 0.586399 | 1.013648 | 0.06248 |
| 214152_at | CCPG1 | -0.22204 | 0.800883 | 0.633218 | 1.012944 | 0.063932 |
| 202686_s_at | AXL | -0.1451 | 0.864932 | 0.741604 | 1.00877 | 0.064499 |
| 206680_at | CD5L | -0.1332 | 0.875288 | 0.759833 | 1.008287 | 0.064949 |
| 204784_s_at | MLF1 | 0.171494 | 1.187077 | 0.989043 | 1.424763 | 0.065527 |
| 206380_s_at | CFP | -0.18619 | 0.830115 | 0.680872 | 1.012072 | 0.065579 |
| 215051_x_at | AIF1 | -0.15806 | 0.853797 | 0.720511 | 1.011739 | 0.06797 |
| 218052_s_at | ATP13A1 | 0.24905 | 1.282806 | 0.981465 | 1.676668 | 0.068301 |
| 227346_at | IKZF1 | -0.34367 | 0.70916 | 0.489198 | 1.028026 | 0.069668 |
| 204510_at | CDC7 | 0.239804 | 1.271 | 0.979934 | 1.648521 | 0.07073 |
| 213537_at | HLA-DPA1 | -0.1053 | 0.900055 | 0.802782 | 1.009114 | 0.071155 |
| 222108_at | AMIGO2 | -0.11505 | 0.891324 | 0.786596 | 1.009995 | 0.07123 |
| 219854_at | ZNF14 | -0.26557 | 0.766768 | 0.573845 | 1.024551 | 0.072504 |
| 222587_s_at | GALNT7 | 0.269922 | 1.309862 | 0.974989 | 1.759752 | 0.073161 |
| 212446_s_at | CERS6 | 0.109739 | 1.115987 | 0.989544 | 1.258588 | 0.073673 |
| 216430_x_at | IGLV1-44 | -0.08114 | 0.922061 | 0.843557 | 1.007871 | 0.073893 |
| 226119_at | PCMTD1 | -0.30708 | 0.735592 | 0.525067 | 1.030527 | 0.074237 |
| 228378_at | C12orf29 | 0.364369 | 1.439606 | 0.964419 | 2.148926 | 0.074634 |
| 218232_at | C1QA | -0.08796 | 0.915802 | 0.831005 | 1.009252 | 0.076032 |
| 200783_s_at | STMN1 | 0.321857 | 1.379687 | 0.966164 | 1.970201 | 0.076627 |
| 203980_at | FABP4 | -0.12595 | 0.881655 | 0.766001 | 1.01477 | 0.079156 |
| 213365_at | ERI2 | 0.317489 | 1.373674 | 0.962961 | 1.959561 | 0.079821 |
| 1552790_a_at | SEC62 | -0.2347 | 0.790811 | 0.608165 | 1.02831 | 0.07984 |
| 214786_at | MAP3K1 | 0.214333 | 1.239036 | 0.973537 | 1.57694 | 0.08151 |
| 204416_x_at | APOC1 | -0.10335 | 0.901809 | 0.80209 | 1.013925 | 0.083869 |
| 239680_at | WDR76 | 0.151577 | 1.163668 | 0.979791 | 1.382053 | 0.084111 |
| 225917_at | ATF7IP | -0.30082 | 0.740212 | 0.525956 | 1.04175 | 0.084461 |
| 210715_s_at | SPINT2 | -0.08102 | 0.922177 | 0.841067 | 1.011109 | 0.08457 |
| 223280_x_at | MS4A6A | -0.12741 | 0.880373 | 0.761666 | 1.017581 | 0.084686 |
| 222663_at | RIOK2 | -0.25239 | 0.776942 | 0.583029 | 1.035351 | 0.084921 |
| 214669_x_at | IGKC | -0.1184 | 0.888344 | 0.776334 | 1.016515 | 0.085113 |
| 208853_s_at | CANX | 0.271801 | 1.312325 | 0.962838 | 1.78867 | 0.085383 |
| 214973_x_at | IGHD | -0.09538 | 0.909029 | 0.815127 | 1.013747 | 0.086433 |
| 225310_at | LOC101928747 | 0.338544 | 1.402903 | 0.95061 | 2.070393 | 0.088215 |
| 235666_at | ITGA8 | -0.12684 | 0.880872 | 0.761209 | 1.019346 | 0.088616 |
| 207194_s_at | ICAM4 | -0.0933 | 0.910917 | 0.81804 | 1.014338 | 0.089035 |
| 223949_at | TMPRSS3 | 0.103023 | 1.108517 | 0.984367 | 1.248325 | 0.089137 |
| 217258_x_at | IGLV1-44 | -0.08647 | 0.917168 | 0.83 | 1.01349 | 0.089701 |
| 209901_x_at | AIF1 | -0.16661 | 0.846528 | 0.69696 | 1.028193 | 0.093021 |
| 57588_at | SLC24A3 | -0.12919 | 0.878808 | 0.755714 | 1.021952 | 0.093361 |
| 205049_s_at | CD79A | -0.09428 | 0.910027 | 0.814976 | 1.016164 | 0.093919 |
| 209189_at | FOS | -0.12263 | 0.884594 | 0.766287 | 1.021168 | 0.094129 |
| 202620_s_at | PLOD2 | 0.086184 | 1.090006 | 0.985329 | 1.205804 | 0.094317 |
| 219607_s_at | MS4A4A | -0.08283 | 0.920504 | 0.835222 | 1.014493 | 0.094942 |
| 201810_s_at | SH3BP5 | -0.13126 | 0.876988 | 0.751689 | 1.023172 | 0.095169 |
| 222285_at | IGHD | -0.09214 | 0.911981 | 0.818198 | 1.016514 | 0.096088 |
| 226977_at | IGIP | -0.23194 | 0.792997 | 0.603145 | 1.042609 | 0.09669 |
| 219208_at | FBXO11 | 0.116422 | 1.12347 | 0.978732 | 1.289612 | 0.098034 |
| 227249_at | NDE1 | 0.289434 | 1.335672 | 0.947604 | 1.882662 | 0.098399 |
| 200838_at | CTSB | -0.21022 | 0.810405 | 0.631504 | 1.039989 | 0.098563 |
| 242281_at | GLUL | -0.13218 | 0.876183 | 0.748126 | 1.026159 | 0.101079 |
| 219777_at | GIMAP6 | -0.11028 | 0.895587 | 0.78487 | 1.021923 | 0.101449 |
| 205548_s_at | BTG3 | 0.263984 | 1.302108 | 0.949346 | 1.785949 | 0.101522 |
| 211908_x_at | IGK | -0.10548 | 0.899896 | 0.792999 | 1.021203 | 0.102096 |
| 228167_at | KLHL6 | -0.24463 | 0.782997 | 0.583576 | 1.050564 | 0.102875 |
| 204044_at | QPRT | -0.17531 | 0.839194 | 0.679737 | 1.036057 | 0.102993 |
| 227718_at | MIR4657 | 0.261309 | 1.298629 | 0.948105 | 1.778745 | 0.103532 |
| 219666_at | MS4A6A | -0.13969 | 0.869631 | 0.733843 | 1.030544 | 0.106826 |
| 204255_s_at | VDR | -0.22866 | 0.795596 | 0.602383 | 1.050782 | 0.107183 |
| 213134_x_at | BTG3 | 0.258204 | 1.294603 | 0.945546 | 1.772517 | 0.10725 |
| 223922_x_at | MS4A6A | -0.18317 | 0.83263 | 0.665942 | 1.041041 | 0.108039 |
| 225621_at | ALG2 | -0.34032 | 0.711544 | 0.469642 | 1.078046 | 0.108396 |
| 205863_at | S100A12 | -0.08263 | 0.920692 | 0.832282 | 1.018494 | 0.108669 |
| 200912_s_at | EIF4A2 | -0.35063 | 0.704245 | 0.457704 | 1.083586 | 0.11075 |
| 213689_x_at | FAM69A | -0.19597 | 0.822035 | 0.646051 | 1.045958 | 0.110848 |
| 217630_at | ANGEL2 | -0.20353 | 0.815846 | 0.634557 | 1.048929 | 0.112423 |
| 202953_at | C1QB | -0.0826 | 0.920723 | 0.831384 | 1.019662 | 0.112724 |
| 219947_at | CLEC4A | -0.14049 | 0.868936 | 0.730014 | 1.034296 | 0.113975 |
| 203476_at | TPBG | 0.113456 | 1.120143 | 0.972406 | 1.290326 | 0.115902 |
| 223423_at | GPR160 | -0.19132 | 0.825867 | 0.650494 | 1.048521 | 0.1162 |
| 201830_s_at | NET1 | 0.124382 | 1.132448 | 0.969566 | 1.322693 | 0.116443 |
| 217138_x_at | IGLC1 | -0.12094 | 0.88609 | 0.761689 | 1.030809 | 0.117152 |
| 214836_x_at | IGK | -0.08427 | 0.919181 | 0.827183 | 1.021411 | 0.117296 |
| 211650_x_at | IGH | -0.06032 | 0.941467 | 0.872873 | 1.01545 | 0.118115 |
| 223502_s_at | TNFSF13B | -0.12468 | 0.882781 | 0.754989 | 1.032203 | 0.118123 |
| 212298_at | NRP1 | -0.08977 | 0.914144 | 0.816775 | 1.02312 | 0.118241 |
| 204249_s_at | LMO2 | -0.12028 | 0.886676 | 0.762457 | 1.031132 | 0.11832 |
| 214768_x_at | IGKC | -0.08247 | 0.920835 | 0.829933 | 1.021693 | 0.119882 |
| 218883_s_at | CENPU | 0.196881 | 1.217599 | 0.949678 | 1.561105 | 0.120482 |
| 225469_at | LYRM5 | -0.29046 | 0.74792 | 0.517753 | 1.080408 | 0.121663 |
| 222530_s_at | MKKS | 0.325718 | 1.385024 | 0.91607 | 2.094046 | 0.122509 |
| 226841_at | MPEG1 | -0.10888 | 0.896834 | 0.780841 | 1.030057 | 0.123346 |
| 234366_x_at | CKAP2 | -0.09542 | 0.90899 | 0.804996 | 1.026417 | 0.123722 |
| 209906_at | C3AR1 | -0.16893 | 0.844571 | 0.680472 | 1.048242 | 0.125391 |
| 203665_at | HMOX1 | -0.09703 | 0.907529 | 0.801612 | 1.02744 | 0.125414 |
| 212158_at | SDC2 | -0.08787 | 0.915879 | 0.81836 | 1.025019 | 0.126077 |
| 221651_x_at | IGK | -0.1681 | 0.845269 | 0.681048 | 1.049089 | 0.127217 |
| 1554057_at | ASH1L-AS1 | 0.23549 | 1.265529 | 0.934967 | 1.712963 | 0.127356 |
| 211643_x_at | IGK | -0.06541 | 0.93668 | 0.861103 | 1.018891 | 0.127518 |
| 220753_s_at | CRYL1 | -0.20958 | 0.810922 | 0.618294 | 1.063561 | 0.129867 |
| 226022_at | SASH1 | -0.13163 | 0.876668 | 0.738557 | 1.040607 | 0.132353 |
| 202524_s_at | SPOCK2 | -0.20001 | 0.818725 | 0.630715 | 1.062778 | 0.132953 |
| 204197_s_at | RUNX3 | 0.258711 | 1.295259 | 0.923375 | 1.816918 | 0.13406 |
| 231121_at | HPS3 | -0.2038 | 0.815625 | 0.624725 | 1.06486 | 0.134125 |
| 204687_at | PARM1 | -0.18396 | 0.831969 | 0.653988 | 1.058388 | 0.13416 |
| 226245_at | KCTD1 | -0.2479 | 0.78044 | 0.563769 | 1.080384 | 0.135176 |
| 49452_at | ACACB | -0.13324 | 0.875258 | 0.734287 | 1.043293 | 0.137024 |
| 203473_at | SLCO2B1 | -0.13108 | 0.877147 | 0.737828 | 1.042774 | 0.137452 |
| 211881_x_at | IGLJ3 | -0.1008 | 0.904114 | 0.791406 | 1.032872 | 0.13785 |
| 231853_at | TUBD1 | -0.26246 | 0.769155 | 0.543679 | 1.088141 | 0.138139 |
| 206210_s_at | CETP | -0.11576 | 0.890686 | 0.763895 | 1.038521 | 0.13953 |
| 236402_at | BRAF | 0.28645 | 1.331692 | 0.90951 | 1.949846 | 0.140909 |
| 202084_s_at | SEC14L1 | -0.30978 | 0.733606 | 0.485686 | 1.108078 | 0.140958 |
| 216365_x_at | CKAP2 | -0.08334 | 0.92004 | 0.823226 | 1.02824 | 0.141816 |
| 203386_at | TBC1D4 | -0.17648 | 0.838212 | 0.661652 | 1.061885 | 0.14363 |
| 227265_at | FGL2 | -0.08221 | 0.921078 | 0.82485 | 1.028531 | 0.144216 |
| 211430_s_at | IGHG1 | -0.13281 | 0.875629 | 0.732155 | 1.047218 | 0.145768 |
| 223287_s_at | FOXP1 | 0.134551 | 1.144023 | 0.954227 | 1.371568 | 0.146018 |
| 227017_at | ERICH1 | 0.216851 | 1.242159 | 0.926735 | 1.664939 | 0.146812 |
| 223660_at | ADORA3 | -0.12245 | 0.884749 | 0.749217 | 1.0448 | 0.148913 |
| 220005_at | P2RY13 | -0.09476 | 0.909587 | 0.799529 | 1.034796 | 0.149825 |
| 209924_at | CCL18 | -0.09984 | 0.904983 | 0.789648 | 1.037163 | 0.151183 |
| 205098_at | CCR1 | 0.088676 | 1.092727 | 0.968027 | 1.23349 | 0.151472 |
| 209200_at | MEF2C | -0.20251 | 0.816679 | 0.618929 | 1.077612 | 0.152269 |
| 208428_at | TAP2 | 0.115074 | 1.121956 | 0.957959 | 1.31403 | 0.153508 |
| 221671_x_at | IGK | -0.15144 | 0.85947 | 0.697728 | 1.058707 | 0.154545 |
| 224391_s_at | SIAE | 0.141087 | 1.151524 | 0.947958 | 1.398805 | 0.155174 |
| 64418_at | SYNRG | -0.28129 | 0.754808 | 0.511366 | 1.114146 | 0.156806 |
| 224795_x_at | IGK | -0.17964 | 0.835567 | 0.651435 | 1.071746 | 0.157239 |
| 236986_at | LINC00582 | -0.17164 | 0.842281 | 0.663202 | 1.069714 | 0.159313 |
| 218805_at | GIMAP1-GIMAP5 | -0.12193 | 0.885207 | 0.746396 | 1.049834 | 0.161174 |
| 226496_at | ZCCHC7 | -0.18793 | 0.828669 | 0.637066 | 1.077899 | 0.161265 |
| 212188_at | KCTD12 | 0.092497 | 1.09691 | 0.963334 | 1.249007 | 0.162673 |
| 205052_at | AUH | -0.2425 | 0.784664 | 0.55757 | 1.104251 | 0.164197 |
| 219355_at | CXorf57 | 0.070893 | 1.073467 | 0.971145 | 1.186569 | 0.165413 |
| 212999_x_at | HLA-DQB1 | -0.07637 | 0.926476 | 0.831492 | 1.032311 | 0.166433 |
| 205632_s_at | PIP5K1B | -0.16739 | 0.845872 | 0.667197 | 1.072397 | 0.166782 |
| 230397_at | SAR1B | 0.106465 | 1.112339 | 0.95645 | 1.293637 | 0.166979 |
| 207734_at | LAX1 | 0.180486 | 1.197799 | 0.927199 | 1.547373 | 0.167148 |
| 225353_s_at | C1QC | -0.07533 | 0.92744 | 0.833415 | 1.032072 | 0.167232 |
| 225140_at | KLF3 | -0.12227 | 0.884909 | 0.743637 | 1.05302 | 0.168264 |
| 1555753_x_at | ERVH-6 | -0.06702 | 0.93518 | 0.850113 | 1.028759 | 0.168429 |
| 206420_at | IGSF6 | -0.11263 | 0.893484 | 0.760146 | 1.050211 | 0.171988 |
| 1564164_at | DENND1B | 0.138775 | 1.148866 | 0.940974 | 1.402688 | 0.173011 |
| 209790_s_at | CASP6 | 0.17256 | 1.188343 | 0.926765 | 1.523752 | 0.173709 |
| 1555349_a_at | ITGB2 | -0.0961 | 0.908376 | 0.790328 | 1.044056 | 0.176064 |
| 203879_at | PIK3CD | -0.14021 | 0.869174 | 0.708732 | 1.065937 | 0.178086 |
| 229997_at | VANGL1 | 0.072366 | 1.075049 | 0.967369 | 1.194715 | 0.178988 |
| 212636_at | QKI | -0.09039 | 0.913574 | 0.800709 | 1.042347 | 0.179108 |
| 207513_s_at | ZNF189 | -0.25169 | 0.777488 | 0.538262 | 1.123036 | 0.179761 |
| 206919_at | ELK4 | 0.251619 | 1.286106 | 0.886029 | 1.866832 | 0.185673 |
| 209385_s_at | PROSC | -0.18342 | 0.832422 | 0.634152 | 1.092684 | 0.186371 |
| 233461_x_at | ZNF226 | 0.149482 | 1.161232 | 0.926343 | 1.455681 | 0.194834 |
| 224964_s_at | GNG2 | -0.06605 | 0.936083 | 0.846885 | 1.034676 | 0.196089 |
| 215621_s_at | IGHD | -0.05022 | 0.951023 | 0.880915 | 1.026711 | 0.198695 |
| 230063_at | ZNF264 | -0.13159 | 0.876702 | 0.716889 | 1.07214 | 0.199995 |
| 204971_at | CSTA | -0.14263 | 0.867072 | 0.697082 | 1.078515 | 0.200164 |
| 213674_x_at | IGHD | -0.03797 | 0.962746 | 0.907864 | 1.020945 | 0.204874 |
| 215214_at | IGLC1 | -0.07958 | 0.923502 | 0.81617 | 1.044949 | 0.206781 |
| 222774_s_at | NETO2 | 0.092337 | 1.096734 | 0.948854 | 1.267661 | 0.211475 |
| 224783_at | UBALD2 | 0.128984 | 1.137672 | 0.929149 | 1.392992 | 0.211813 |
| 241819_at | TNFSF8 | -0.05599 | 0.945547 | 0.865787 | 1.032655 | 0.213021 |
| 200703_at | DYNLL1 | 0.323289 | 1.381664 | 0.829003 | 2.302763 | 0.214818 |
| 211798_x_at | IGLJ3 | -0.08004 | 0.923082 | 0.812945 | 1.048141 | 0.216957 |
| 228181_at | SLC30A1 | 0.148134 | 1.159668 | 0.915167 | 1.469491 | 0.220133 |
| 227647_at | KCNE3 | -0.13128 | 0.876968 | 0.709423 | 1.084084 | 0.22489 |
| 216510_x_at | IGHA1 | -0.05837 | 0.943301 | 0.858423 | 1.036572 | 0.225008 |
| 217988_at | CCNB1IP1 | 0.168873 | 1.18397 | 0.898172 | 1.560708 | 0.230892 |
| 214460_at | LSAMP | -0.07159 | 0.930911 | 0.827788 | 1.046881 | 0.232036 |
| 212681_at | EPB41L3 | -0.06826 | 0.934017 | 0.834892 | 1.04491 | 0.233063 |
| 216044_x_at | FAM69A | -0.15216 | 0.858848 | 0.668235 | 1.103832 | 0.23467 |
| 203562_at | FEZ1 | -0.11366 | 0.892558 | 0.73892 | 1.078141 | 0.238268 |
| 51158_at | FAM174B | -0.11591 | 0.890553 | 0.734221 | 1.080173 | 0.239228 |
| 222783_s_at | SMOC1 | 0.098346 | 1.103344 | 0.936321 | 1.300161 | 0.240272 |
| 225520_at | LOC100996643 | 0.134284 | 1.143717 | 0.913936 | 1.431271 | 0.240595 |
| 217853_at | TNS3 | -0.14926 | 0.861343 | 0.670459 | 1.106573 | 0.24292 |
| 210244_at | CAMP | -0.08513 | 0.918394 | 0.795564 | 1.060188 | 0.245193 |
| 225893_at | RC3H1 | 0.269629 | 1.309478 | 0.830775 | 2.064016 | 0.245482 |
| 224837_at | FOXP1 | 0.121278 | 1.128939 | 0.918549 | 1.387519 | 0.249093 |
| 229551_x_at | ZNF367 | 0.120985 | 1.128608 | 0.918399 | 1.386932 | 0.249942 |
| 229437_at | MIR155 | -0.04478 | 0.956211 | 0.884638 | 1.033574 | 0.259304 |
| 213388_at | LOC100996724 | -0.14893 | 0.861627 | 0.664704 | 1.116889 | 0.260609 |
| 204787_at | VSIG4 | -0.13811 | 0.871005 | 0.684405 | 1.108479 | 0.261552 |
| 226456_at | RMI2 | 0.101589 | 1.106929 | 0.925694 | 1.323647 | 0.265454 |
| 225171_at | ARHGAP18 | -0.14942 | 0.86121 | 0.66112 | 1.121857 | 0.268033 |
| 229435_at | GLIS3 | -0.04816 | 0.952977 | 0.874897 | 1.038025 | 0.269463 |
| 220595_at | PDZRN4 | 0.081336 | 1.084736 | 0.938054 | 1.254354 | 0.272523 |
| 209708_at | MOXD1 | -0.08618 | 0.91743 | 0.786284 | 1.070449 | 0.273528 |
| 201278_at | DAB2 | -0.07053 | 0.931899 | 0.82086 | 1.057958 | 0.275893 |
| 201506_at | TGFBI | -0.06375 | 0.938244 | 0.835993 | 1.053002 | 0.278923 |
| 1555476_at | IREB2 | -0.10219 | 0.902861 | 0.749794 | 1.087176 | 0.280981 |
| 223620_at | GPR34 | -0.10353 | 0.901648 | 0.746494 | 1.089049 | 0.282568 |
| 216560_x_at | IGLC1 | -0.03448 | 0.966103 | 0.907002 | 1.029056 | 0.284312 |
| 235735_at | TNFSF8 | -0.06308 | 0.938864 | 0.83632 | 1.053982 | 0.285057 |
| 225853_at | GNPNAT1 | 0.163372 | 1.177475 | 0.872655 | 1.588769 | 0.285152 |
| 235507_at | PCMTD1 | -0.18109 | 0.834358 | 0.59762 | 1.164876 | 0.287504 |
| 206631_at | PTGER2 | -0.07407 | 0.928609 | 0.810102 | 1.064453 | 0.287649 |
| 234339_s_at | GLTSCR2 | -0.09093 | 0.913077 | 0.771562 | 1.080548 | 0.289898 |
| 201441_at | COX6B1 | 0.166225 | 1.180839 | 0.867625 | 1.607124 | 0.290503 |
| 203066_at | CHST15 | -0.24982 | 0.778941 | 0.489945 | 1.238403 | 0.290937 |
| 229491_at | SLC9B2 | 0.177388 | 1.194094 | 0.857614 | 1.662589 | 0.293531 |
| 201455_s_at | NPEPPS | 0.222958 | 1.249768 | 0.823356 | 1.897016 | 0.295044 |
| 212900_at | SEC24A | -0.16721 | 0.846019 | 0.617797 | 1.158548 | 0.297193 |
| 230836_at | ST8SIA4 | -0.12534 | 0.882198 | 0.69473 | 1.120253 | 0.303798 |
| 43427_at | ACACB | -0.08708 | 0.916607 | 0.775971 | 1.082732 | 0.305535 |
| 202080_s_at | TRAK1 | 0.10184 | 1.107206 | 0.910933 | 1.345769 | 0.306334 |
| 222692_s_at | FNDC3B | -0.14296 | 0.866787 | 0.65819 | 1.141494 | 0.308774 |
| 214230_at | CDC42 | 0.11381 | 1.120539 | 0.898862 | 1.396886 | 0.311575 |
| 213820_s_at | STARD5 | 0.099458 | 1.104572 | 0.909018 | 1.342196 | 0.317098 |
| 204749_at | NAP1L3 | 0.054472 | 1.055983 | 0.948957 | 1.17508 | 0.317768 |
| 208771_s_at | LOC101928830 | -0.15776 | 0.854055 | 0.62502 | 1.167019 | 0.321997 |
| 222867_s_at | MED31 | -0.1433 | 0.86649 | 0.651433 | 1.152543 | 0.324838 |
| 201218_at | CTBP2 | -0.06071 | 0.941095 | 0.832885 | 1.063365 | 0.329986 |
| 204020_at | PURA | -0.12492 | 0.882569 | 0.684239 | 1.138385 | 0.336091 |
| 230391_at | CD84 | -0.08353 | 0.919861 | 0.774808 | 1.09207 | 0.340063 |
| 238034_at | CANX | -0.13708 | 0.8719 | 0.657718 | 1.15583 | 0.340548 |
| 222478_at | VPS36 | -0.15976 | 0.852348 | 0.612837 | 1.185466 | 0.34254 |
| 221210_s_at | NPL | -0.07677 | 0.926098 | 0.79032 | 1.085204 | 0.342562 |
| 228897_at | DERL3 | -0.11216 | 0.8939 | 0.70903 | 1.126973 | 0.342726 |
| 211644_x_at | IGK | -0.0342 | 0.966375 | 0.900162 | 1.037459 | 0.34492 |
| 204949_at | ICAM3 | -0.09105 | 0.912972 | 0.755447 | 1.103345 | 0.346076 |
| 1555745_a_at | LYZ | -0.06452 | 0.937515 | 0.819457 | 1.072581 | 0.347418 |
| 1557309_at | DENND1B | -0.12807 | 0.879789 | 0.673356 | 1.14951 | 0.347882 |
| 204928_s_at | SLC10A3 | 0.137884 | 1.147842 | 0.86016 | 1.531741 | 0.348932 |
| 218615_s_at | TMEM39A | -0.18985 | 0.827082 | 0.554612 | 1.233412 | 0.351801 |
| 224413_s_at | TM2D2 | -0.17544 | 0.839088 | 0.578741 | 1.216553 | 0.35461 |
| 208146_s_at | CPVL | -0.05013 | 0.951108 | 0.855161 | 1.05782 | 0.355526 |
| 204602_at | DKK1 | 0.053236 | 1.054679 | 0.941881 | 1.180985 | 0.356292 |
| 218723_s_at | RGCC | -0.05639 | 0.945169 | 0.837411 | 1.066794 | 0.361213 |
| 200628_s_at | WARS | 0.081498 | 1.084911 | 0.910699 | 1.292448 | 0.361481 |
| 225123_at | SESN3 | -0.08861 | 0.915199 | 0.756167 | 1.107678 | 0.362882 |
| 211505_s_at | STAU1 | -0.18465 | 0.831393 | 0.558481 | 1.237668 | 0.363034 |
| 201106_at | GPX4 | -0.14646 | 0.863757 | 0.629169 | 1.185812 | 0.365003 |
| 201413_at | HSD17B4 | -0.1801 | 0.835184 | 0.56449 | 1.235687 | 0.367525 |
| 213093_at | PRKCA | -0.0779 | 0.925057 | 0.780724 | 1.096073 | 0.368088 |
| 207857_at | LILRA2 | 0.067583 | 1.069919 | 0.921028 | 1.242879 | 0.376714 |
| 204084_s_at | CLN5 | -0.13836 | 0.870781 | 0.640397 | 1.184046 | 0.377514 |
| 202760_s_at | AKAP2 | 0.118257 | 1.125533 | 0.865031 | 1.464486 | 0.378609 |
| 204137_at | GPR137B | 0.049983 | 1.051254 | 0.939556 | 1.17623 | 0.383146 |
| 218324_s_at | SPATS2 | -0.17792 | 0.837008 | 0.56091 | 1.249011 | 0.383643 |
| 228518_at | IGHG1 | -0.03718 | 0.963505 | 0.886107 | 1.047664 | 0.384222 |
| 209662_at | CETN3 | -0.146 | 0.864155 | 0.620639 | 1.203218 | 0.387298 |
| 235458_at | HAVCR2 | -0.0748 | 0.927925 | 0.782907 | 1.099805 | 0.388273 |
| 237177_at | CNTN4 | -0.0668 | 0.935381 | 0.803647 | 1.088708 | 0.388387 |
| 206641_at | TNFRSF17 | -0.17666 | 0.838063 | 0.556653 | 1.261737 | 0.397405 |
| 225681_at | CTHRC1 | 0.039787 | 1.040589 | 0.948901 | 1.141136 | 0.397868 |
| 236782_at | SAMD3 | -0.04469 | 0.956293 | 0.861527 | 1.061483 | 0.401271 |
| 216541_x_at | IGHG1 | -0.03873 | 0.96201 | 0.878602 | 1.053337 | 0.402596 |
| 211640_x_at | IGHG1 | -0.03179 | 0.968707 | 0.898928 | 1.043902 | 0.404543 |
| 241869_at | APOL6 | -0.07378 | 0.928875 | 0.780528 | 1.105418 | 0.405941 |
| 210613_s_at | SYNGR1 | 0.059928 | 1.06176 | 0.920914 | 1.224148 | 0.409189 |
| 232044_at | RBBP6 | -0.14229 | 0.867366 | 0.617243 | 1.218845 | 0.412334 |
| 214770_at | MSR1 | 0.054241 | 1.055738 | 0.926055 | 1.203583 | 0.417287 |
| 232382_s_at | PCMTD1 | -0.10162 | 0.903374 | 0.705607 | 1.15657 | 0.420185 |
| 213325_at | PVRL3 | 0.068664 | 1.071076 | 0.905644 | 1.266728 | 0.422468 |
| 226591_at | PWAR6 | 0.069264 | 1.071719 | 0.904791 | 1.269445 | 0.422674 |
| 211795_s_at | FYB | -0.07644 | 0.926412 | 0.768062 | 1.117407 | 0.424159 |
| 236958_at | LOC100506473 | 0.103162 | 1.108671 | 0.86073 | 1.428034 | 0.424433 |
| 223405_at | NPL | -0.09976 | 0.905057 | 0.706584 | 1.159278 | 0.429638 |
| 226710_at | C8orf82 | 0.118407 | 1.125703 | 0.838187 | 1.511841 | 0.431339 |
| 208636_at | ACTN1 | -0.0621 | 0.939789 | 0.803448 | 1.099265 | 0.437439 |
| 221584_s_at | KCNMA1 | -0.05598 | 0.945558 | 0.819043 | 1.091617 | 0.44496 |
| 222991_s_at | UBQLN1 | 0.175229 | 1.191519 | 0.759085 | 1.870301 | 0.446219 |
| 242939_at | TFDP1 | 0.055521 | 1.057092 | 0.915594 | 1.220456 | 0.448899 |
| 207325_x_at | MAGEA1 | 0.052038 | 1.053416 | 0.919779 | 1.20647 | 0.452156 |
| 216517_at | IGKC | -0.03906 | 0.961689 | 0.868269 | 1.065162 | 0.453721 |
| 220993_s_at | GPR63 | 0.079949 | 1.083232 | 0.878467 | 1.335726 | 0.454542 |
| 227133_at | FAM199X | 0.151473 | 1.163546 | 0.780301 | 1.735022 | 0.457456 |
| 224800_at | WDFY1 | 0.111939 | 1.118445 | 0.830827 | 1.505632 | 0.460495 |
| 1558279_a_at | KDSR | -0.10127 | 0.903687 | 0.690041 | 1.183481 | 0.461806 |
| 1559336_at | LOC101928505 | -0.06832 | 0.933964 | 0.778161 | 1.120962 | 0.463147 |
| 210232_at | CDC42 | -0.09265 | 0.911509 | 0.711125 | 1.168359 | 0.464473 |
| 1552354_at | C19orf26 | -0.07713 | 0.925769 | 0.751442 | 1.140537 | 0.468695 |
| 214070_s_at | ATP10B | 0.034777 | 1.035388 | 0.941406 | 1.138753 | 0.473808 |
| 217865_at | RNF130 | -0.02714 | 0.973221 | 0.90265 | 1.049309 | 0.479723 |
| 209921_at | SLC7A11 | -0.06875 | 0.933559 | 0.771158 | 1.130161 | 0.480758 |
| 218454_at | PLBD1 | -0.05023 | 0.95101 | 0.827051 | 1.093547 | 0.480843 |
| 202672_s_at | ATF3 | -0.07091 | 0.931543 | 0.763665 | 1.136327 | 0.484283 |
| 1552280_at | TIMD4 | -0.04677 | 0.954309 | 0.836803 | 1.088316 | 0.485434 |
| 229305_at | CENPU | 0.111945 | 1.118451 | 0.815325 | 1.534276 | 0.487632 |
| 211796_s_at | TRBC1 | -0.05354 | 0.947864 | 0.811927 | 1.106561 | 0.497815 |
| 217682_at | RP11-473I1.9 | 0.089393 | 1.093511 | 0.843791 | 1.417134 | 0.499141 |
| 224441_s_at | USP45 | 0.057132 | 1.058796 | 0.895552 | 1.251795 | 0.503666 |
| 204057_at | IRF8 | -0.0699 | 0.932485 | 0.758347 | 1.14661 | 0.507468 |
| 230102_at | ETV5 | 0.044893 | 1.045916 | 0.915812 | 1.194503 | 0.507729 |
| 226069_at | PRICKLE1 | -0.08483 | 0.918671 | 0.714538 | 1.181121 | 0.508216 |
| 219551_at | EAF2 | 0.094748 | 1.099382 | 0.829642 | 1.456821 | 0.509465 |
| 219492_at | CHIC2 | -0.17532 | 0.83919 | 0.497613 | 1.415236 | 0.510862 |
| 225493_at | CCNT1 | 0.168661 | 1.183719 | 0.715418 | 1.958561 | 0.511516 |
| 228336_at | PWWP2A | -0.17402 | 0.840282 | 0.499505 | 1.413547 | 0.511986 |
| 201987_at | MED13 | -0.14705 | 0.863248 | 0.555063 | 1.342545 | 0.513988 |
| 201582_at | SEC23B | -0.13759 | 0.871455 | 0.571074 | 1.329835 | 0.523436 |
| 218888_s_at | NETO2 | 0.059098 | 1.060879 | 0.882401 | 1.275457 | 0.529475 |
| 228772_at | HNMT | -0.03962 | 0.961156 | 0.849421 | 1.087588 | 0.529777 |
| 230793_at | LRRC16A | -0.07061 | 0.93183 | 0.744869 | 1.165716 | 0.53661 |
| 219159_s_at | SLAMF7 | 0.076692 | 1.079709 | 0.845906 | 1.378135 | 0.537935 |
| 236835_at | FUT8-AS1 | -0.05302 | 0.948365 | 0.801002 | 1.12284 | 0.538362 |
| 201279_s_at | DAB2 | -0.04076 | 0.960061 | 0.84258 | 1.093922 | 0.540525 |
| 1568665_at | RNF103 | 0.087989 | 1.091976 | 0.821572 | 1.451379 | 0.544437 |
| 201324_at | EMP1 | -0.04707 | 0.954024 | 0.818544 | 1.111929 | 0.546982 |
| 208762_at | SUMO1 | 0.053634 | 1.055098 | 0.885839 | 1.256698 | 0.547714 |
| 211634_x_at | IGHM | -0.02392 | 0.976362 | 0.902641 | 1.056104 | 0.550367 |
| 227084_at | DTNA | 0.067572 | 1.069907 | 0.855311 | 1.338345 | 0.554112 |
| 211635_x_at | IGHA1 | -0.02541 | 0.97491 | 0.894611 | 1.062416 | 0.562317 |
| 242028_at | ZNF709 | 0.067361 | 1.069682 | 0.851114 | 1.344378 | 0.563523 |
| 235736_at | SMKR1 | 0.051938 | 1.05331 | 0.880866 | 1.259514 | 0.569105 |
| 219064_at | ITIH5 | -0.0469 | 0.95418 | 0.809402 | 1.124853 | 0.576401 |
| 235306_at | GIMAP8 | -0.0716 | 0.930906 | 0.721567 | 1.200977 | 0.581713 |
| 226236_at | LINC00493 | -0.0899 | 0.914019 | 0.663588 | 1.258962 | 0.582099 |
| 223195_s_at | SESN2 | 0.056928 | 1.058579 | 0.864028 | 1.296936 | 0.582713 |
| 219243_at | GIMAP4 | -0.04298 | 0.957929 | 0.820073 | 1.11896 | 0.587705 |
| 210757_x_at | DAB2 | -0.03925 | 0.961514 | 0.831963 | 1.111237 | 0.595057 |
| 212007_at | UBXN4 | -0.06916 | 0.933178 | 0.722225 | 1.205748 | 0.596835 |
| 204438_at | MRC1 | -0.04386 | 0.957086 | 0.811026 | 1.129452 | 0.603659 |
| 204820_s_at | BTN3A2 | -0.08058 | 0.922581 | 0.678349 | 1.254747 | 0.607543 |
| 214257_s_at | SEC22B | -0.08278 | 0.920551 | 0.670658 | 1.263558 | 0.608445 |
| 215498_s_at | LOC100996792 | -0.08188 | 0.921384 | 0.673002 | 1.261435 | 0.609442 |
| 212671_s_at | HLA-DQA1 | -0.02875 | 0.971658 | 0.869626 | 1.08566 | 0.611486 |
| 203528_at | SEMA4D | -0.06453 | 0.937509 | 0.730071 | 1.203887 | 0.613046 |
| 215043_s_at | GUSBP3 | -0.03855 | 0.962181 | 0.828512 | 1.117416 | 0.613426 |
| 222930_s_at | AGMAT | 0.035545 | 1.036184 | 0.902113 | 1.19018 | 0.615112 |
| 201280_s_at | DAB2 | -0.03216 | 0.968356 | 0.852224 | 1.100313 | 0.621772 |
| 203988_s_at | FUT8 | 0.032811 | 1.033355 | 0.905971 | 1.178651 | 0.624973 |
| 209822_s_at | VLDLR | -0.05367 | 0.947747 | 0.76286 | 1.177444 | 0.627889 |
| 208814_at | HSPA4 | -0.04207 | 0.958805 | 0.807438 | 1.13855 | 0.631329 |
| 231894_at | SARS | 0.06121 | 1.063122 | 0.824729 | 1.370425 | 0.636579 |
| 205917_at | ZNF264 | -0.09426 | 0.910049 | 0.6152 | 1.34621 | 0.637058 |
| 222533_at | CRBN | -0.08795 | 0.915805 | 0.63521 | 1.320349 | 0.637507 |
| 202878_s_at | CD93 | -0.05066 | 0.950597 | 0.769924 | 1.173667 | 0.637588 |
| 241703_at | RUNDC3B | 0.026992 | 1.02736 | 0.917341 | 1.150573 | 0.640455 |
| 223553_s_at | DOK3 | 0.05483 | 1.056361 | 0.837432 | 1.332524 | 0.643565 |
| 224897_at | WDR26 | -0.10815 | 0.897494 | 0.562508 | 1.431971 | 0.650046 |
| 238440_at | CLYBL | 0.045101 | 1.046134 | 0.858753 | 1.274401 | 0.654253 |
| 214265_at | ITGA8 | -0.03317 | 0.967376 | 0.833959 | 1.122136 | 0.661346 |
| 225422_at | CDC26 | 0.08777 | 1.091737 | 0.735284 | 1.620992 | 0.663406 |
| 200003_s_at | MIR6805 | 0.087547 | 1.091494 | 0.734525 | 1.621945 | 0.664854 |
| 203217_s_at | ST3GAL5 | -0.06754 | 0.934689 | 0.686678 | 1.272276 | 0.667695 |
| 226879_at | HVCN1 | 0.03436 | 1.034958 | 0.882161 | 1.21422 | 0.673326 |
| 201236_s_at | BTG2 | -0.08256 | 0.920752 | 0.622996 | 1.36082 | 0.6787 |
| 1554474_a_at | MOXD1 | -0.03038 | 0.970075 | 0.837589 | 1.123519 | 0.685109 |
| 216438_s_at | TMSB4X | -0.022 | 0.978241 | 0.87835 | 1.089493 | 0.688933 |
| 213831_at | HLA-DQA1 | -0.03082 | 0.969654 | 0.833066 | 1.128635 | 0.690763 |
| 207638_at | TMPRSS15 | 0.03246 | 1.032993 | 0.879542 | 1.213215 | 0.69239 |
| 225923_at | VAPB | -0.05729 | 0.944324 | 0.709163 | 1.257466 | 0.695018 |
| 1560397_s_at | KLHL6 | -0.02331 | 0.976959 | 0.869277 | 1.097979 | 0.695627 |
| 201397_at | PHGDH | -0.03195 | 0.96856 | 0.821141 | 1.142444 | 0.704541 |
| 226084_at | MAP1B | 0.019043 | 1.019226 | 0.920928 | 1.128015 | 0.712851 |
| 237097_at | EHD4 | 0.040223 | 1.041043 | 0.838302 | 1.292816 | 0.71588 |
| 214428_x_at | C4A | -0.02508 | 0.975229 | 0.85174 | 1.116622 | 0.716523 |
| 209803_s_at | PHLDA2 | -0.02031 | 0.979899 | 0.877584 | 1.094143 | 0.718181 |
| 225706_at | GLCCI1 | -0.04574 | 0.955287 | 0.744292 | 1.226096 | 0.719424 |
| 206170_at | ADRB2 | 0.049701 | 1.050956 | 0.799206 | 1.382008 | 0.722044 |
| 212155_at | RNF187 | -0.04889 | 0.952282 | 0.726767 | 1.247775 | 0.722895 |
| 217678_at | SLC7A11 | 0.030495 | 1.030965 | 0.867273 | 1.225552 | 0.729572 |
| 222752_s_at | TMEM206 | -0.07837 | 0.924625 | 0.592158 | 1.443757 | 0.730332 |
| 222309_at | C6orf62 | -0.04738 | 0.953728 | 0.723223 | 1.257699 | 0.737148 |
| 225685_at | CDC42EP3 | 0.041263 | 1.042126 | 0.816938 | 1.329387 | 0.739745 |
| 213984_at | PDS5A | -0.05753 | 0.944092 | 0.66716 | 1.335975 | 0.745349 |
| 1554309_at | EIF4G3 | 0.025106 | 1.025423 | 0.878493 | 1.196928 | 0.750355 |
| 223501_at | TNFSF13B | -0.03667 | 0.963997 | 0.765974 | 1.213215 | 0.754631 |
| 229360_at | ZNF280B | -0.0322 | 0.968317 | 0.78383 | 1.196226 | 0.765288 |
| 213624_at | SMPDL3A | -0.01855 | 0.981622 | 0.86583 | 1.112899 | 0.772086 |
| 213005_s_at | KANK1 | -0.0335 | 0.967056 | 0.770788 | 1.213302 | 0.772251 |
| 223003_at | C19orf43 | -0.0601 | 0.94167 | 0.623631 | 1.421902 | 0.774999 |
| 203349_s_at | ETV5 | -0.02908 | 0.971343 | 0.792901 | 1.189942 | 0.778899 |
| 210813_s_at | XRCC4 | -0.0371 | 0.963584 | 0.735898 | 1.261715 | 0.787379 |
| 202972_s_at | FAM13A | -0.02801 | 0.972378 | 0.790387 | 1.196272 | 0.791057 |
| 204050_s_at | CLTA | 0.0475 | 1.048646 | 0.735419 | 1.495282 | 0.793024 |
| 229492_at | VANGL1 | 0.014619 | 1.014727 | 0.908481 | 1.133397 | 0.795582 |
| 238066_at | RBP7 | -0.02065 | 0.979566 | 0.835672 | 1.148236 | 0.798953 |
| 235479_at | CPEB2 | -0.05311 | 0.948274 | 0.625918 | 1.436647 | 0.802137 |
| 1554719_at | NDUFA10 | 0.021712 | 1.02195 | 0.851157 | 1.227014 | 0.815989 |
| 33494_at | ETFDH | -0.03103 | 0.969444 | 0.743781 | 1.263574 | 0.818451 |
| 1568817_at | AL133493.2 | 0.020767 | 1.020984 | 0.849826 | 1.226613 | 0.824453 |
| 1556361_s_at | ANKRD13C | -0.02006 | 0.980144 | 0.819958 | 1.171625 | 0.825652 |
| 214844_s_at | DOK5 | 0.020992 | 1.021214 | 0.840012 | 1.241505 | 0.833171 |
| 220354_at | MCF2L-AS1 | -0.01498 | 0.985132 | 0.856531 | 1.133043 | 0.833764 |
| 223008_s_at | TMEM245 | -0.04669 | 0.954387 | 0.610575 | 1.491799 | 0.837686 |
| 221880_s_at | FAM174B | -0.01625 | 0.983886 | 0.841666 | 1.150137 | 0.838403 |
| 211637_x_at | IGHV3-23 | -0.00894 | 0.991095 | 0.899837 | 1.091608 | 0.855985 |
| 210078_s_at | KCNAB1 | -0.01716 | 0.98299 | 0.816492 | 1.18344 | 0.856219 |
| 201286_at | SDC1 | -0.02285 | 0.977411 | 0.763267 | 1.251637 | 0.856305 |
| 215949_x_at | IGHM | 0.007158 | 1.007184 | 0.931817 | 1.088645 | 0.856855 |
| 202990_at | PYGL | 0.023107 | 1.023376 | 0.795302 | 1.316857 | 0.857452 |
| 226342_at | SPTBN1 | -0.01146 | 0.988606 | 0.863677 | 1.131605 | 0.867955 |
| 229844_at | FOXP1 | 0.013573 | 1.013665 | 0.85981 | 1.19505 | 0.871622 |
| 210587_at | INHBE | 0.006842 | 1.006865 | 0.919895 | 1.102057 | 0.881998 |
| 208451_s_at | C4A | 0.005774 | 1.005791 | 0.92782 | 1.090313 | 0.888467 |
| 239363_at | INIP | -0.01773 | 0.982428 | 0.7634 | 1.264298 | 0.890437 |
| 207076_s_at | ASS1 | -0.00637 | 0.993645 | 0.905384 | 1.090511 | 0.893149 |
| 218974_at | SOBP | -0.01577 | 0.984354 | 0.779034 | 1.243787 | 0.894884 |
| 205220_at | HCAR3 | -0.00933 | 0.990711 | 0.860991 | 1.139975 | 0.896301 |
| 205071_x_at | XRCC4 | -0.00822 | 0.991818 | 0.875175 | 1.124009 | 0.897602 |
| 205119_s_at | FPR1 | 0.009636 | 1.009682 | 0.87142 | 1.169882 | 0.897957 |
| 227525_at | GLCCI1 | -0.0152 | 0.984917 | 0.778001 | 1.246864 | 0.899486 |
| 240834_at | OTULIN | -0.01025 | 0.989798 | 0.837795 | 1.16938 | 0.904053 |
| 207132_x_at | PFDN5 | -0.02365 | 0.976627 | 0.655948 | 1.454079 | 0.907288 |
| 211434_s_at | CCRL2 | -0.00679 | 0.993237 | 0.88271 | 1.117602 | 0.910231 |
| 231182_at | WIPF1 | 0.011809 | 1.011879 | 0.820163 | 1.24841 | 0.912262 |
| 219247_s_at | ZDHHC14 | -0.00924 | 0.990806 | 0.837411 | 1.172299 | 0.914288 |
| 218231_at | NAGK | -0.01412 | 0.985982 | 0.758299 | 1.282027 | 0.916071 |
| 201847_at | LIPA | -0.01289 | 0.987191 | 0.768475 | 1.268155 | 0.919642 |
| 220431_at | TMPRSS11E | -0.00565 | 0.994367 | 0.888002 | 1.113472 | 0.922037 |
| 212233_at | MAP1B | 0.011303 | 1.011367 | 0.800547 | 1.277706 | 0.924497 |
| 242100_at | CHSY3 | -0.00505 | 0.994965 | 0.891553 | 1.110372 | 0.928169 |
| 225362_at | FAM122B | 0.012041 | 1.012113 | 0.76882 | 1.332397 | 0.931598 |
| 1568672_at | EAF2 | -0.00819 | 0.991843 | 0.813542 | 1.20922 | 0.935432 |
| 208436_s_at | IRF7 | -0.01008 | 0.989973 | 0.774272 | 1.265764 | 0.93594 |
| 201141_at | GPNMB | 0.005555 | 1.00557 | 0.848392 | 1.191869 | 0.948925 |
| 212192_at | KCTD12 | -0.00296 | 0.997046 | 0.909468 | 1.093058 | 0.94972 |
| 201195_s_at | SLC7A5 | 0.004539 | 1.004549 | 0.845722 | 1.193205 | 0.958775 |
| 228099_at | ZNF550 | -0.00985 | 0.990201 | 0.665873 | 1.4725 | 0.961208 |
| 215285_s_at | PHTF1 | 0.007253 | 1.007279 | 0.721961 | 1.405355 | 0.965953 |
| 210908_s_at | PFDN5 | -0.00761 | 0.99242 | 0.677117 | 1.454545 | 0.968884 |
| 208056_s_at | CBFA2T3 | 0.006324 | 1.006344 | 0.729595 | 1.388068 | 0.969255 |
| 213194_at | ROBO1 | 0.001294 | 1.001294 | 0.911644 | 1.099761 | 0.978436 |
| 1565660_at | FUT6 | 0.001843 | 1.001845 | 0.86364 | 1.162165 | 0.980587 |
| 209286_at | CDC42EP3 | 0.003383 | 1.003389 | 0.763477 | 1.318691 | 0.980639 |
| 227542_at | SOCS6 | -0.00113 | 0.998869 | 0.897718 | 1.111418 | 0.983429 |
| 228370_at | SNORD116-4 | -0.00258 | 0.997421 | 0.773802 | 1.285664 | 0.984095 |
| 226489_at | TMCC3 | 0.001443 | 1.001444 | 0.838368 | 1.19624 | 0.987307 |
| 227917_at | LOC100506990 | -0.00164 | 0.998358 | 0.753146 | 1.323408 | 0.990885 |
| 226006_at | PET100 | -0.00105 | 0.99895 | 0.692251 | 1.441532 | 0.995522 |
