# Supplementary material for: Development and Validation of a 9-Gene Prognostic Signature in Patients With Multiple Myeloma
Source: Front Oncol. 2019 Jan 8;8:615. doi: 10.3389/fonc.2018.00615 (PMC6331463; doi:10.3389/fonc.2018.00615)
Supplement: Supplementary Table 2 — The 9 hub genes with non-zero coefficients in the LASSO Cox proportional hazards regression model. [file Table_2.docx]

**Supplementary table 2** The 9 hub genes with non-zero coefficients in the LASSO Cox proportional hazards regression model.

| Probe | gene | Coefficient |
| --- | --- | --- |
| 201137_s_at | HLA.DPB1 | -0.00987 |
| 201291_s_at | TOP2A | 0.057615 |
| 202345_s_at | FABP5 | 0.070558 |
| 202437_s_at | CYP1B1 | -0.02076 |
| 209374_s_at | IGHM | -0.05666 |
| 213008_at | FANCI | 0.01564 |
| 213975_s_at | LYZ | -0.01653 |
| 221606_s_at | HMGN5 | 0.05666 |
| 231175_at | BEND6 | -0.01576 |
