# Supplementary material for: Development and Validation of a 9-Gene Prognostic Signature in Patients With Multiple Myeloma
Source: Front Oncol. 2019 Jan 8;8:615. doi: 10.3389/fonc.2018.00615 (PMC6331463; doi:10.3389/fonc.2018.00615)
Supplement: Supplementary Table 3 — Cox proportional hazards regression analysis the association between the clinical characteristics and overall survival of multiple myeloma in the training set. [file Table_3.docx]

**Supplementary table 3** Cox proportional hazards regression analysis the association between the clinical characteristics and overall survival of multiple myeloma in the training set.

| Variable | Univariate analysis | | | |  | | Multivariable analysis | | | |
| --- | --- | --- | --- | --- | --- | --- | --- | --- | --- | --- |
|  | HR | LCI | UCI | P value |  | HR | | LCI | UCI | P value |
| Age | 1.0183 | 0.9969 | 1.0402 | 0.0943 |  | 1.0089 | | 0.9869 | 1.0313 | 0.4320 |
| B2M | 1.0804 | 1.0591 | 1.1022 | <0.001 |  | 1.0873 | | 1.0561 | 1.1195 | <0.001 |
| CRP | 1.0010 | 0.9947 | 1.0074 | 0.7528 |  | 0.9932 | | 0.9827 | 1.0038 | 0.2077 |
| LDH | 1.0074 | 1.0053 | 1.0094 | <0.001 |  | 1.0048 | | 1.0025 | 1.0072 | 0.0001 |
| BMPC | 1.0144 | 1.0066 | 1.0223 | 0.0003 |  | 1.0013 | | 0.9926 | 1.0100 | 0.7739 |
| MRI | 1.0244 | 1.0136 | 1.0353 | <0.001 |  | 1.0214 | | 1.0088 | 1.0340 | 0.0008 |
| 9-gene signature | 0.2664 | 0.1772 | 0.4007 | <0.001 |  | 0.3868 | | 0.2462 | 0.6078 | <0.001 |

**Abbreviations:** B2M, β2-microglobin; CRP, C-reaction protein; LDH, lactate dehydrogenase; BMPC, bone marrow plasma cell; MRI, magnetic resonance imaging.

**Supplementary table 4** Cox proportional hazards regression analysis on the association between the clinical characteristics and overall survival of multiple myeloma patients in the test set.

|  | | Univariate analysis | | | |  | Multivariable analysis | | | |
| --- | --- | --- | --- | --- | --- | --- | --- | --- | --- | --- |
| Variable | HR | | LCI | UCI | P value |  | HR | LCI | UCI | P value |
| Age | 1.0358 | | 1.0082 | 1.0641 | 0.0107 |  | 1.0296 | 1.0017 | 1.0582 | 0.0373 |
| B2M | 1.0953 | | 1.0517 | 1.1406 | <0.001 |  | 1.0778 | 1.0272 | 1.1308 | 0.0023 |
| CRP | 1.0197 | | 1.0068 | 1.0329 | 0.0027 |  | 1.0162 | 1.0019 | 1.0306 | 0.0262 |
| LDH | 1.0040 | | 1.0010 | 1.0070 | 0.0089 |  | 1.0033 | 1.0000 | 1.0067 | 0.0512 |
| BMPC | 1.0025 | | 0.9935 | 1.0116 | 0.5883 |  | 0.9941 | 0.9835 | 1.0048 | 0.2802 |
| MRI | 1.0049 | | 0.9895 | 1.0207 | 0.5344 |  | 0.9953 | 0.9778 | 1.0130 | 0.5992 |
| 9-gene signature | 0.5115 | | 0.3137 | 0.8339 | 0.0072 |  | 0.5301 | 0.3081 | 0.9122 | 0.0219 |

**Abbreviations:** B2M, β2-microglobin; CRP, C-reaction protein; LDH, lactate dehydrogenase; BMPC, bone marrow plasma cell; MRI, magnetic resonance imaging.

**Supplementary table 5** Cox proportional hazards regression analysis on the association between the clinical characteristics and event-free survival of multiple myeloma patients in the training set.

|  | | Univariate analysis | | | |  | Multivariable analysis | | |  |
| --- | --- | --- | --- | --- | --- | --- | --- | --- | --- | --- |
| Variable | HR | | LCI | UCI | P value |  | HR | LCI | UCI | P value |
| Age | 1.0137 | | 0.9963 | 1.0314 | 0.1237 |  | 1.0014 | 0.9836 | 1.0196 | 0.8756 |
| B2M | 1.0684 | | 1.0492 | 1.0879 | <0.001 |  | 1.0786 | 1.0515 | 1.1065 | <0.001 |
| CRP | 1.0039 | | 0.9991 | 1.0086 | 0.1136 |  | 0.9958 | 0.9880 | 1.0036 | 0.2938 |
| LDH | 1.0067 | | 1.0048 | 1.0086 | <0.001 |  | 1.0049 | 1.0027 | 1.0072 | <0.001 |
| BMPC | 1.0117 | | 1.0054 | 1.0179 | 0.0002 |  | 1.0010 | 0.9940 | 1.0081 | 0.7764 |
| MRI | 1.0164 | | 1.0068 | 1.0261 | 0.0008 |  | 1.0107 | 0.9997 | 1.0217 | 0.0556 |
| 9-gene signature | 0.3321 | | 0.2395 | 0.4606 | <0.001 |  | 0.4662 | 0.3202 | 0.6789 | 0.0001 |

**Abbreviations:** B2M, β2-microglobin; CRP, C-reaction protein; LDH, lactate dehydrogenase; BMPC, bone marrow plasma cell; MRI, magnetic resonance imaging.

**Supplementary table 6** Cox proportional hazards regression analysis on the association between the clinical characteristics and event-free survival of multiple myeloma patients in the test set.

|  | | Univariate analysis | | | |  | Multivariable analysis | | | |
| --- | --- | --- | --- | --- | --- | --- | --- | --- | --- | --- |
| Variable | HR | | LCI | UCI | P value |  | HR | LCI | UCI | P value |
| Age | 1.0135 | | 0.9917 | 1.0359 | 0.2264 |  | 1.0047 | 0.9818 | 1.0280 | 0.6917 |
| B2M | 1.0916 | | 1.0492 | 1.1356 | <0.001 |  | 1.0879 | 1.0369 | 1.1415 | 0.0006 |
| CRP | 1.0155 | | 1.0033 | 1.0278 | 0.0125 |  | 1.0138 | 1.0008 | 1.0270 | 0.0369 |
| LDH | 1.0026 | | 0.9997 | 1.0055 | 0.0766 |  | 1.0024 | 0.9992 | 1.0056 | 0.1466 |
| BMPC | 1.0044 | | 0.9965 | 1.0123 | 0.2753 |  | 0.9937 | 0.9843 | 1.0032 | 0.1919 |
| MRI | 1.0000 | | 0.9865 | 1.0137 | 0.9955 |  | 0.9892 | 0.9739 | 1.0047 | 0.1712 |
| 9-gene signature | 0.5174 | | 0.3447 | 0.7765 | 0.0015 |  | 0.4598 | 0.2909 | 0.7268 | 0.0009 |

**Abbreviations:** B2M, β2-microglobin; CRP, C-reaction protein; LDH, lactate dehydrogenase; BMPC, bone marrow plasma cell; MRI, magnetic resonance imaging.

**Supplementary table 7** The prognostic role of the 9-gene signature in the independent validation cohort.

|  | Univariate analysis | | | | | Multivariable analysis | | | |
| --- | --- | --- | --- | --- | --- | --- | --- | --- | --- |
|  | HR | LCI | UCI | P value |  | HR | LCI | UCI | P value |
| 9-gene signature | 10.6091 | 3.2120 | 35.0409 | 0.0001 |  | 14.8092 | 1.2282 | 178.5591 | 0.0339 |
| Age | 1.0329 | 1.0099 | 1.0563 | 0.0048 |  | 1.0518 | 0.9941 | 1.1129 | 0.0795 |
| Gender | 1.0741 | 0.7072 | 1.6314 | 0.7375 |  | 1.4478 | 0.6261 | 3.3481 | 0.3870 |
| ISS Stage II | 1.9879 | 1.0560 | 3.7421 | 0.0333 |  | 1.2536 | 0.4525 | 3.4730 | 0.6637 |
| ISS stage III | 2.5806 | 1.4322 | 4.6498 | 0.0016 |  | 1.4519 | 0.4998 | 4.2172 | 0.4931 |
| Treatment line | 1.1224 | 0.9685 | 1.3007 | 0.1250 |  | 1.2508 | 0.9544 | 1.6393 | 0.1048 |
| 17p13d | 2.5308 | 1.3490 | 4.7479 | 0.0038 |  | 2.9605 | 1.0179 | 8.6102 | 0.0463 |
| Translocation t(4:14) | 0.9614 | 0.5192 | 1.7804 | 0.9004 |  | 0.5293 | 0.2314 | 1.2109 | 0.1319 |
| 1q21 gain | 1.6970 | 1.0681 | 2.6963 | 0.0252 |  | 2.2983 | 1.0249 | 5.1540 | 0.0434 |
